# Functional Neuroanatomy of the Noradrenergic Locus Coeruleus: Its Roles in the Regulation of Arousal and Autonomic Function Part II: Physiological and Pharmacological Manipulations and Pathological Alterations of Locus Coeruleus Activity in Humans

**DOI:** 10.2174/157015908785777193

**Published:** 2008-09

**Authors:** E. R Samuels, E Szabadi

**Affiliations:** Psychopharmacology Section, University of Nottingham, Division of Psychiatry, Queen’s Medical Centre, Nottingham NG7 2UH, UK

**Keywords:** Locus coeruleus, arousal, autonomic function, noxious stimuli, anxiety, Parkinson’s disease, Alzheimer’s disease, aging.

## Abstract

The locus coeruleus (LC), the major noradrenergic nucleus of the brain, gives rise to fibres innervating most structures of the neuraxis. Recent advances in neuroscience have helped to unravel the neuronal circuitry controlling a number of physiological functions in which the LC plays a central role. Two such functions are the regulation of arousal and autonomic activity, which are inseparably linked largely *via* the involvement of the LC. Alterations in LC activity due to physiological or pharmacological manipulations or pathological processes can lead to distinct patterns of change in arousal and autonomic function. Physiological manipulations considered here include the presentation of noxious or anxiety-provoking stimuli and extremes in ambient temperature. The modification of LC-controlled functions by drug administration is discussed in detail, including drugs which directly modify the activity of LC neurones (e.g., *via* autoreceptors, storage, reuptake) or have an indirect effect through modulating excitatory or inhibitory inputs. The early vulnerability of the LC to the ageing process and to neurodegenerative disease (Parkinson’s and Alzheimer’s diseases) is of considerable clinical significance. In general, physiological manipulations and the administration of stimulant drugs, α_2_-adrenoceptor antagonists and noradrenaline uptake inhibitors increase LC activity and thus cause heightened arousal and activation of the sympathetic nervous system. In contrast, the administration of sedative drugs, including α_2_-adrenoceptor agonists, and pathological changes in LC function in neurodegenerative disorders and ageing reduce LC activity and result in sedation and activation of the parasympathetic nervous system.

## 1 BRIEF OUTLINE OF THE ROLE OF THE LOCUS COERULEUS IN THE MAINTENANCE OF AROUSAL AND REGULATION OF AUTONOMIC FUNCTIONS

The LC, the largest group of noradrenergic neurones in the central nervous system, is a major nucleus involved in the neural pathways controlling arousal and autonomic function. These physiological functions are inseparably linked, largely due to the central role of the LC in controlling these functions. The LC projects extensively to widespread areas of the neuraxis (see Part I) and these projections can result in both excitatory effects *via* the activation of α_1_-adrenoceptors and inhibitory effects *via* the stimulation of α_2_-adrenoceptors [206]. Therefore, complex changes in the neural circuitry underlying arousal and autonomic function result from changes in LC activity.

### 1.1 Arousal

The LC is recognised as a major wakefulness-promoting nucleus [304, 305], where the activity of the LC closely correlates with level of arousal [16, 17, 18, 122, 123, 355, 360]. This wakefulness-promoting action results from the dense projections from the LC to most areas of the cerebral cortex [208] and from the multitude of projections from the LC to alertness-modulating nuclei (see Part I). The LC exerts an excitatory influence on wakefulness-promoting neurones such as cholinergic neurones of the BF [111, 126, 203, 205] and of the PPT and LDT nuclei [26], cortically-projecting neurones of the thalamus [280, 281] and serotonergic neurones of the DR [219, 309, 375], and an inhibitory influence on sleep-promoting GABA-ergic neurones of the BF [268, 288, 451] and VLPO of the hypothalamus [74, 288, 319]. Thus, increases in LC activity result in increases in EEG signs of alertness [29] whilst inactivation of the LC reduces this EEG activity [30, 91], demonstrating a reduction in alertness. Furthermore, the LC exerts a powerful inhibitory influence on REM sleep, probably by inhibiting a subgroup of cholinergic neurones in the pedunculopontine tegmental nucleus involved in REM sleep [185] (see Part I). Indeed, electrical stimulation of the LC has been found to reduce the quantity of SWS and REM sleep in a human subject [211], demonstrating an increase in wakefulness. A schematic diagram outlining the sleep/arosal neuronal network, highlighting the central position of the LC, is shown in Fig. (**1**).

### 1.2 Autonomic Functions

It is also well recognised that the LC plays an important role in controlling autonomic functions (see Part I). As a major premotor autonomic nucleus, the LC sends direct projections to the sympathetic preganglionic neurones in the spinal cord [208, 316, 489] and parasympathetic preganglionic neurones in the brainstem and spinal cord (*EWN*: [50, 255]; *salivatory nuclei*: [203, 419]; *DMV*: [309, 457, 490]; *nucleus ambiguus*: [208, 490]; *spinal cord*: [208, 385, 490, 506]). It should be noted that the DMV also contains somatomotor neurones to varying degrees in different mammalian species [256], and in fact in humans it is almost completely somatmotor. [52]. On the other hand, the EWN is an almost exclusively preganglonic parasympathetic nucleus in man [52]. In general, the LC increases sympathetic activity *via* the activation of α_1_-adrenoceptors on preganglionic sympathetic neurones [248] and reduces parasympathetic activity *via* the activation of α_2_-adrenoceptors on preganglionic parasympathetic neurones [418, 465, 501]. Furthermore, the LC also exerts an indirect effect on autonomic activity *via* projections to other premotor autonomic nuclei such as the PVN [207, 208, 309, 440, 461], the RVLM [470], and the CR [174, 208]. It is of interest that while the influence of the LC on premotor autonomic neurones in the PVN and CR is excitatory, it is inhibitory on neurones in the RVLM (see Fig. (**2**)). Finally, the LC may modulate autonomic activity by projections to the cerebral cortex and amygdala [208, 293], structures which are known to influence the activity of premotor sympathetic neurones in the PVN [173, 420] and RVLM [437]. The projections of the LC to the amygdala [90, 218] and to the PVN [381, 430] have both been linked to the autonomic response to stress, consisting of generalised sympathetic activation. A schematic diagram outlining the autonomic neuronal network, highlighting the central position of the LC, is shown in Fig. (**2**).

The activation of the LC results in a well-defined pattern of autonomic changes: in tissues receiving a predominantly sympathetic innervation (e.g., arterioles and sweat glands) there is an increase in activity, whereas in those receiving predominantly parasympathetic innervation (e.g., salivary glands) there is a decrease in activity. It should be noted that the pressor effect observed after LC activation is attenuated by the inhibition of the RVLM [470], resulting in only moderate increases in heart rate and blood pressure [412, 482]. Furthermore, in a tissue receiving a dual sympathetic/parasympathetic innervation (e.g., iris), where the two autonomic inputs have opposing effects, the effect of LC activation on sympathetic activity is enhanced by the inhibition of the parasympathetic output. The consequences of LC activation are, therefore, observable as an increase in pupil diameter [341, 342], attenuation of the light reflex response [22, 38, 40], moderate increases in heart rate and blood pressure [106, 154, 251, 412, 429, 482], suppression of the baroreflex response [192, 401], and a reduction in salivation [446]. The modulation of the baroreflex response by the LC may reflect to some extent the activity of central adrenergic neurones which have been shown to project to the LC (see Section 3.1.4.5 in Part I) and to be sensitve to changes in blood pressure [252].

### 1.3 Correlation between Arousal and Autonomic Function

The central role of the LC in the combined regulation of arousal and autonomic function is highlighted by the association between the level of arousal in pupillary activity. It has been shown that changes in the level of arousal are associated with changes in pupil diameter [186, 254] and spontaneous pupillary fluctuations in darkness [257, 507], and there is evidence that the pupillary changes directly reflect LC activity [354]. Indeed, the close relationship between level of arousal and pupil diameter has been exploited in the PST, which records pupil diameter over a period of eleven minutes. During the period of recording slow fluctuations appear in the diameter of the pupil (“pupillary fatigue waves”), and it has been shown that the power of these fluctuations can be used as a reliable index of the level of arousal [493]. As there is a close parallelism between fluctuations in the firing rates of LC neurones and fluctuations in pupil diameter (see Fig. (**3**)), it is likely that the pupillary fatigue waves represent fluctuations in LC activity, and thus may provide a direct physiological indicator of the functioning of this important brainstem nucleus.

### 1.4 Scope of the Review

The activity of the LC can be altered through a variety of physiological manipulations such as the presentation of a noxious or anxiety-provoking stimulus or by variations in ambient temperature, through pharmacological manipulations such as the administration of drugs which act directly at the autoreceptors located on the neurones of the LC, that modify the reuptake or storage of noradrenaline, or that act indirectly to modify LC activity, through neuronal loss from the LC in aging, and through pathological changes that occur as a result of neurodegenerative disease (PD, AD) and brainstem trauma resulting in coma. In this review we attempt to delineate the effects of experimental manipulations and pathological changes on LC activity and hence on level of arousal and autonomic function.

## 2 PHYSIOLOGICAL MANIPULATION OF LOCUS COERULEUS ACTIVITY

LC neurone activity is determined by a number of varied inputs, as described in our companion paper (see Part I). Physiological manipulations, such as the presentation of a noxious or anxiety-provoking stimulus or an extreme in ambient temperature, can modulate the strength of these inputs, and therefore the extent of excitation or inhibition of the noradrenergic neurones, to alter the overall activity of the LC. The effect on LC activity resulting from these manipulations can often be detected *via* the measurement of resting pupil diameter and/or pupillary reflexes to sudden light stimuli, since the LC is so integral to pupillary control [444]; see above and Part I. For changes in cortical activity following LC activation, see Section 2.1.1. in Part I.

### 2.1 Noxious Stimuli

The LC is innervated by the sensory neurones of the dorsal horn of the spinal cord: this innervation provides a method of communicating nociceptive information to this nucleus [61, 82, 309]; see 3.1.6, Part I. Neurone activity within the LC is increased following the presentation of a noxious stimulus, measurable as an increase in electrical activity [109, 124, 220, 361, 412] and an increase in c-fos expression [56, 326, 327, 340, 394, 462, 478]; see [324] for a review. The pattern of LC neurone firing after the presentation of a noxious stimulus involves an initial burst of activity followed by a period of quiescence [420]. Interestingly, microinjection of the α_2_-adrenoceptor antagonist idazoxan into the LC, which is likely to result in an increase in LC activity (see section 3.2, below), has been reported to increase the responsiveness of LC neurones to noxious stimulation [420]. In addition, noradrenaline levels in both the LC and PVN, a projection area of the LC (see 2.2.2.2, part I), are increased following noxious stimulation [324, 374, 411], presumably as a result of an increase in noradrenaline synthesis in LC neurones. Indeed, the PVN has been implicated in pain perception. Interestingly, the increase in LC activity following the presentation of a noxious stimulus has been found to inhibit neurones of the basolateral nucleus of the amygdala and this inhibition may be involved in the formation of emotional memories following a noxious event [70].

It is expected that an increase in LC activity following noxious stimulation would, in general, increase sympathetic and decrease parasympathetic activity (see section 1.2, above), and this may be related to the paradigm of *pupillary reflex dilatation*, the best studied autonomic response to noxious stimulation. Pupillary reflex dilatation is observable in both experimental animals [177, 231] and human subjects [66, 237, 481, 504].

Interestingly, drugs that are known to modify LC activity (see 3.2, below) also affect pupillary reflex dilatation in a way consistent with the alteration in LC activity. Thus, manipulations which decrease LC activity in experimental animals (e.g., monoamine depletion by reserpine and alpha-methyl-para-tyrosine) antagonise pupillary reflex dilatation [175]. However, paradoxically, both an α_2_-adrenoceptor antagonist, yohimbine [175, 177] and an α_2_-adrenoceptor agonist, dexmedetomidine [237] have been reported to inhibit pupillary reflex dilatation. As the observations with yohimbine were made in cats and those with dexmedetomidine in humans, these findings can be reconciled by a well-documented species difference in the pupillary effects of drugs interacting with α_2_-adrenoceptors. It has been shown that agonists constrict and antagonists dilate the pupil in man but evoke effects in the opposite direction in cats, indicating the preferential action of these drugs at pre-synaptic α_2_-adrenoceptors in man and post-synaptic α_2_-adrenoceptors in the cat (see 3.2, below). In both man and the cat, the administration of an α_2_-adrenoceptor antagonist is expected to activate the LC and increase its sensitivity to further activation by noxious stimulation [409], which would be expected to lead to facilitation of pupillary reflex dilatation. However, in the cat this effect is likely to be masked by miosis, resulting from the activation of the EWN as a result of the blockade of post-synaptic inhibitory α_2_-adrenoceptors, leading to the apparent attenuation of pupillary reflex dilatation. On the other hand, the α_2_-adrenoceptor agonist is expected to reduce LC activity and thus attenuate the LC activation resulting from the noxious stimulation, manifesting as an apparent reduction in pupillary reflex dilatation. Indeed, this effect has been reported in man [237]. It should be noted that Larson and Talke [237] interpreted this finding on the basis of a postulated anti-nociceptive effect of dexmedetomidine, since they assumed that the LC had been completely “switched off” by the pre-treatment of their subjects with opiates.

A well-studied experimental paradigm of sympathetic activation by noxious stimulation in human subjects is the cold pressor test, evoked by a painful cold stimulus to the hand, which leads to both an increase in blood pressure and to pupil dilatation [454, 455]. This response has two interesting features. Firstly, it is not accompanied by a reduction in light reflex amplitude [455], as would be expected on the basis of increased LC activity, which could be predicted to lead to enhanced noradrenergic inhibition of the EWN. This observation raises the possibility that different populations of LC neurones may be responsible for mediating the sympathetic activating and parasympathetic inhibiting effects of the LC on pre-ganglionic autonomic neurones (see 5, below), where the cold pressor test may influence only the sympathetic pre-autonomic neurones in the LC. Secondly, while the increase in blood pressure evoked by the cold pressor test can be antagonised by diazepam, the increase in pupil diameter is resistant to it [189]. This observation may indicate that different structures may be involved in mediating the autonomic effects of the cold pressor test, the pupillary effect is likely to be mediated by the LC whereas the pressor effect by the PVN. Furthermore, while GABA_A_ receptors occur in both the LC [71, 216, 314] and the PVN [158, 162, 212, 467], those in the LC are insensitive to diazepam [71], whereas those in the PVN, like GABA_A_ receptors in general, probably are sensitive to this drug.

### 2.2 Anxiety

Anxiety is generally defined as an emotional state evoked by threatening stimuli, although pharmacological intervention can also produce increased anxiety levels [443]. It is known that the amygdala is critical to the generation of anxiety and anxious responses [90, 218, 242] and there is a two-way excitatory connection between the amygdala and the LC (see Part I). It, therefore, follows that LC activation could be expected to lead to anxiety through the activation of the amygdala and, conversely, anxiety producing stimuli that increase the activity of the amygdala could be expected to lead to LC activation. This circular argument makes it difficult to determine in a given situation whether anxiety is produced by the activation of the LC or whether the LC is activated as a result of increased anxiety. In certain situations, however, the two processes can be delineated (see below).

#### 2.2.1 Anxiogenic Effect of LC Activation

Electrical stimulation of the LC evokes fear-related behavioural responses [365], which can be alleviated by clonidine, morphine, and diazepam [365], drugs known to suppress LC activity (see sections 3.1 and 3.2). Administration of the α_2_-adrenoceptor antagonist yohimbine activates the LC (see section 3.2), which in turn produces an increase in anxiety [141, 282, 300, 453]. Conversely, bilateral lesions of the LC [363] or of the ascending noradrenergic projection arising from it [475], lead to a reduction in fear-related responses.

#### 2.2.2 LC Activation by States of Anxiety

It is well recognized that patients suffering from different forms of anxiety disorder show evidence of altered autonomic regulation [443]. Many of the changes reported are consistent with increased LC activity, leading to enhancement of sympathetic discharge and inhibition of parasympathetically mediated functions. Thus, it has been reported that anxious patients show hypersensitivity of their eccrine sweat glands to intradermally injected muscarinic cholinoceptor agonists, such as carbachol, a finding indicating increased sympathetic activity. Eccrine sweat glands receive a cholinergic sympathetic innervation (see Fig. (**2**)), and their activity, and also their sensitivity to cholinoceptor agonists, is modulated by impulse traffic in the sympathetic fibres, high firing rates being associated with high, and low firing rates with low sweat gland activity/sensitivity [443]. Furthermore, it has been shown that patients suffering from generalized anxiety disorder have attenuated light reflexes [22], consistent with increased noradrenergic inhibition of the EWN originating from the LC.

Experimental situations of induced fear increase anxiety *via* the activation of the amygdala and lead to LC activation [68, 69, 85, 360, 364]. The experimental paradigm of conditioned fear can be used to produce states of fear and anxiety in the laboratory and thus can be used to assess the modulation of the LC during these states. Fear conditioning, where a neutral stimulus (CS) is temporally associated with an aversive stimulus (US) and thus gains an aversive character itself, produces an increase in LC activity as demonstrated by increased c-fos expression when the CS is presented alone [195, 253]. In addition, rats selectively bred to demonstrate high levels of anxiety show increased c-fos expression within the LC [376] and in human volunteers subliminal fear signals have been found to elicit activity within the LC, as detected by functional neuroimaging [250].

The increase in LC activity following the conditioned fear paradigm is accompanied by an increase in arousal and sympathetic function. For example, REM sleep has been observed to concomitantly decrease in response to the presentation of a fear conditioned stimulus [253] and this is consistent with the activation of the LC and the expected increase in arousal. The activation of the sympathetic nervous system by conditioned fear results in a variety of changes in autonomic activity and in particular there are two measurable changes in the pupil: an increase in resting pupil diameter and a reduction in the amplitude of the light reflex response [36, 38, 39, 40, 42]. Furthermore, it has been reported that patients suffering from generalised anxiety disorder have attenuated light reflex responses [22]. These pupillary effects are likely to reflect the dual influence of LC activation on the pupil: the increase in resting pupil diameter may suggest an increase in sympathetic outflow and/or a decrease in parasympathetic outflow to the iris, whereas the decrease in light reflex amplitude is the result of inhibition of the parasympathetic outflow to the iris (see above). Interestingly, of the two pupillary effects of conditioned fear, evoked by the threat of a mild electric shock, only the reduction in light reflex response amplitude was related quantatively to the degree of subjective anxiety, whereas the increase in pupil diameter was not [42], and only the reduction in light reflex response amplitude was susceptible to antagonism by the anxiolytic drug diazepam, whereas the increase in pupil diameter was not [36, 40]. These observations suggest that conditioned fear may preferentially activate the pre-parasympathetic neurones in the LC *via* the amygdala, leading to the anxiety-dependent reduction in light reflex response amplitude, whereas it may cause some indirect activation of the presympathetic neurones *via* the arousal system, resulting in some increase in pupil diameter. The differential effects of diazepam on the two components of the pupillary anxiety response may reflect the fact that diazepam may increase the GABAergic inhibition of amygdala neurones, thus leading to the attenuation of the fear-induced activation of pre-parasympathetic LC neurones, whereas it may not be able to modify the activity of pre-sympathetic LC neurones which do not contain diazepam-sensitive GABA receptors [71]; see also Section 3 and Fig. (**6**). 

Stress exposure, which may also be classed as an anxiety-provoking situation, is also associated with enhanced LC activity [124] and with increased noradrenaline release [51, 453, 101, 322, 323], following the presentation of a stressful stimulus in experimental animals, such as the rat and cat Indeed, chronic stress exposure produces long-term alterations in LC firing and noradrenaline release [51, 398, 321, 322]. For reviews on the activation of the central noradrenergic system by exposure to stressful stimuli, see refs. 324, 328; see also Section 2.1.

The enhancement of LC activity by stressful stimuli can be detected by changes observed in autonomic activity. Tasks inducing stress *via* high levels of cognitive load have been found to increase pupil diameter and reduce light reflex amplitude compared to no or low cognitive load tasks [426, 427]. Furthermore, these studies have identified both an increase in sympathetic activity and a decrease in parasympathetic activity in the modulation of pupil diameter following the application of a cognitive load, consistent with LC activation. Recently it has been shown that remembering emotionally-loaded material results in activation of the LC in humans, as detected by functional magnetic resonance imaging (fMRI), together with an increase in pupil diameter [428].

#### 2.2.3 Role of the LC in Mediating Fear Responses

It has been proposed that an increase in LC activity mediates the behavioural responses associated with fear and anxiety [67], and this is supported by observations associating the increased firing of the LC following the presentation of fear-inducing stimuli with behavioural manifestations of fear [51]. For example, α_2_-adrenoceptor knockout mice lacking the physiological brake of LC activity (see section 3.2) show both an increase in LC activity and an increase in freezing responses in the conditioned fear paradigm [92], whilst lesions of the LC attenuate the freezing response both to the unconditioned aversive stimulus and to the conditioned cue [306]. 

An interesting possibility that results from the interaction of the amygdala and the LC (see above) is that activation of the LC may contribute to the fear-potentiated startle response. The startle response involves rapid involuntary contractions of facial and skeletal musculature in response to a sudden intense stimulus, for example, a loud sound, and this reaction is measurable in man as an EMG response from the orbicularis oculi muscle (eyeblink startle response). The LC is likely to be involved in modulating the startle response since the LC projects to the motoneurones in the facial nucleus [208]; see 2.3.5.1, Part I, which innervates the orbicularis oculi muscle. This projection is likely to exert a facilitatory influence on the motoneurones as demonstrated by the enhancement of motoneurone activity evoked by the extracellular microiontophoretic application of noradrenaline to the facial motoneurones [359, 471, 491]. The administration of an α_2_-adrenoceptor agonist, which reduces LC activity, has been found to reduce the startle response [3, 4, 233, 379], whereas the α_2_-adrenoceptor antagonist yohimbine, which is known to enhance LC activity [200, 413, 453], enhances the amplitude of the acoustic startle response [294]. It is possible that this modulation of the startle response arises from the withdrawal or potentiation of the excitatory input to the orbicularis oculi muscle from the LC, leading to a reduction or enhancement in the tone of this muscle respectively [379]. Indeed, the wakefulness-promoting drug modafinil, assumed to act through enhancing the activity of the LC [184, 383]; see also 3.2.1.2, was found to antagonise the reduction in startle response amplitude found following clonidine administration [379].

This startle response can be potentiated by the threat of an electric shock, inducing a state of fear *via* the activation of the amygdala [93]. This paradigm is therefore used as an important laboratory model of anxiety, both in animals [301, 369, 408, 498] and humans [11, 144, 147, 210]. The LC may contribute to the activation of the startle pathway in the fear-potentiated startle paradigm in two ways. Firstly, the acoustic stimulus itself may prime motoneurone activity *via* the LC: the acoustic stimulus leads to the activation of the caudal pontine reticular nucleus, the major pre-motor integrator of the startle response [226], which activates not only the facial motoneurones but also the LC [379], which in turn results in the facilitation of motoneurone activity (see above). Secondly, the activation of the amygdala by fear-conditioning may increase LC activity (see above) and thus the noradrenergic output of this nucleus. In this way, the dual excitatory input to the LC from the caudal pontine reticular nucleus and the amygdala enhances the tone of the motoneurones of the facial nucleus and contributes to the fear-potentiated startle response. Interestingly, the administration of the α_2_-adrenoceptor agonist clonidine, which reduces LC activity, has been found to result in a reduction in the fear-potentiated startle response [94], whilst the administration of the α_2_ adrenoceptor antagonist yohimbine, which is known to enhance LC activity, enhances the fear-potentiated startle response [94].

### 2.3 Ambient Temperature

Preoptic nuclei of the hypothalamus, including the VLPO, are intimately involved in temperature regulation [108, 387], and neurones in these areas change their activities in response both to increases and decreases in body temperature [108]. Body temperature in turn may be influenced by ambient temperature [15]. Furthermore, neurones in the VLPO project profusely to the LC (see 3.1.3.1, Part I). Therefore, shifts in ambient temperature induce changes in LC activity *via* the mediation of the anterior hypothalamus. 

Acute and chronic modulation of ambient temperature has different effects on LC activity, acute temperature changes increasing and chronic exposure decreasing LC activity. The increase in LC activity in response to acute cold stress has been demonstrated as increases in c-fos expression [223, 508] and in the activity of the rate-limiting enzyme in noradrenaline synthesis, tyrosine hydroxylase [72, 320]. Similarly, acute high ambient temperature increases LC neurone activity [295]. Activation of the LC by high ambient temperature (40°C) is associated with activation of the sympathetic nervous system, manifesting as increases in body temperature, heart rate, carbachol-evoked sweating, and physiological finger tremor [15] and a reduction in the recovery time of the pupillary light reflex response [247]. Some of these sympathetic responses (increases in body temperature and heart rate) could be antagonised by clonidine [15] consistent with the involvement of the LC. In contrast, warm ambient temperature (33°C) has no effect on LC neurone firing [223]. It is of interest to note that acute thermal cutaneous stimulation also activates neurones of the LC, *via* the release of excitatory amino acids from the nucleus paragigantocellularis [153].

Chronic cold stress has been found to reduce LC neurone activity, measurable as a reduction in tyrosine hydroxylase activity and an increase in α_2_-adrenoceptor mRNA expression in the LC [113]. Similarly, chronic high ambient temperature reduces LC activity, measurable as a reduction in tyrosine hydroxylase activity [130].

## 3 PHARMACOLOGICAL ALTERATIONS OF LOCUS COERULEUS ACTIVITY

Drugs can modify LC activity either directly by interacting with different aspects of the function of the noradrenergic neurones, i.e. by modifying firing rate and/or release *via* an action at inhibitory autoreceptors, modifying the storage of the neurotransmitter in presynaptic vesicles, and interfering with the elimination of synaptically released noradrenaline *via* the reuptake mechanism. Drugs may also have an indirect effect on LC activity *via* the modification of the activities of excitatory or inhibitory inputs to the LC. 

### 3.1 Drugs with a Direct Action on LC Activity

#### 3.1.1 Drugs Acting at Autoreceptors

LC neurone activity can be modulated *via* the administration of drugs that stimulate or block the inhibitory autoreceptors located presynaptically on the LC neurones (see 3.2, Part I). Autoreceptors occur both on the cell body (somatodendritic autoreceptors) and at the nerve ending (terminal autoreceptors). The somatodendritic autoreceptors modulate the firing rate of the neurone whereas the terminal autoreceptors modulate the release of the neurotransmitter. Exposure to the α_2_-adrenoceptor agonist clonidine has been used to activate these autoreceptors, whilst the α_2_-adrenoceptor antagonist yohimbine has been used to block them. Administration of clonidine to the LC hyperpolarises the neuronal membrane *via* an increase in potassium conductance leading to a reduction of the spontaneous firing rate of the neurones, resulting in the inhibition of the LC [5, 6, 114, 273, 303, 438, 439, 494, 496]. The inhibition of LC activity by clonidine is accompanied by a fall in plasma noradrenaline concentration [182]. Other α_2_-adrenoceptor agonists such as dexmedetomidine have a similar effect on LC activity [73, 209]. In contrast, administration of yohimbine to the LC increases neuronal firing [200, 300, 303, 413, 453] and noradrenaline release [487]. Other α_2_-adrenoceptor antagonists (BRL44408, RS79948, RX821002) have also been found to have an excitatory influence on the LC, observable as an increase in noradrenaline release at LC terminal regions [115].

Noradrenergic pathways arising from the LC may mediate either excitatory effects, *via* the activation of α_1_-adrenoceptors, on the target cells (ie. in the cerebral cortex and IML) or inhibitory effects, *via* the activation of α_2_-adrenoceptors located on the target cells (eg. in the VLPO, EWN, RVLM) (see Figs. (**2**) and (**4**)). Since the inhibitory effect is mediated by the same post-synaptic receptors that also attenuate the activity of LC neurones in a presynaptic location, i.e., α_2_-adrenoceptors, the net effect of a α_2_-adrenoceptor agonist depends on the relative activation of pre- and post-synaptic receptors (see Table **1**). We wish to consider the effect of clonidine on seven noradrenergic pathways, five inhibitory and two excitatory, whose roles in the regulation of four functions (arousal, pupil control, blood pressure, salivation) have been extensively studied (see Fig. (**4**) and Table **1**). There are five sites where clonidine interacts with inhibitory α_2_-adrenoceptors: LC (autoreceptors; 1), VLPO (2), EWN (3), RVLM (4) and salivatory nuclei (5). The “switching off” of LC activity by clonidine *via* activation of inhibitory autoreceptors results in attenuation of both the excitatory (stimulatory) effects and the inhibitory effects (disinhibition) mediated by noradrenergic pathways arising from the LC. On the other hand, activation of post-synaptic inhibitory α_2_-adrenoceptors results in potentiation of the noradrenergic inhibition of the target cells in the VLPO, EWN, RVLM and salivatory nuclei. The net effect of clonidine on an inhibitory noradrenergic pathway therefore will reflect the balance between the results of the opposing pre-synaptic and post-synaptic effects: if the pre-synaptic effect predominates, the result will be attenuation of the function and if the post-synaptic effect predominates, the effect will be the augmentation of the function. α_2_-Adrenoceptor antagonists (e.g., yohimbine) exert effects on the four functions studied which are opposite to those of the agonists, reflecting the blockade of both autoreceptors and post-synaptic receptors.

##### 3.1.1.1 Arousal

α_2_-Adrenoceptor agonists have potent sedative effects due to the activation of the autoreceptors on LC neurones resulting in the “switching off” of the activity of these neurones. The reduction in LC activity leads to the attenuation of the noradrenergic stimulation of the cerebral cortex and disinhibition of the VLPO which in turn leads to increased inhibition of the wakefulness-promoting histaminergic pathways originating from the tuberomamillary nucleus (TMN; see 1.1 above and 2.2.2.1 in Part I). It should be noted, however, that α_2_-adrenoceptor agonists also activate inhibitory post-synaptic α_2_-adrenoceptors in the VLPO, which is expected to lead to an increase in the level of arousal. This effect usually is masked by the presynaptic effect of these drugs on LC neurones resulting in a sedative effect. However, in some species (cats, rats, and mice) the stimulant effect of α_2._-adrenoceptor agonists has been observed (see 3.1.1.6, below). The sedative effect of clonidine in humans is well demonstrated in the laboratory [182, 184, 296, 342, 379, 421, 486], and it is a known drawback to the clinical use of this drug as an anti-hypertensive agent [45, 261]. Other α_2_-adrenoceptor agonists, for example dexmedetomidine, have also been shown to have pronounced sleep-promoting effects [269, 388], and this drug is used clinically as an anaesthetic. Interestingly, the opioid analgesic tramadol also possesses α_2_-adrenoceptor stimulating activity and completely inhibits LC activity [31], and has been shown to be associated with sedation as a side effect [243, 249]. These drugs have been shown to promote SWS [99]. In contrast to clonidine, the α_2_-adrenoceptor antagonist yohimbine has been shown to possess some alerting effects [342], and the increase in wakefulness has been demonstrated to be associated with cortical desynchronisation [99].

##### 3.1.1.2 Pupil Control

α_2_-Adrenoceptor activation in the LC leads to a reduction in sympathetically-mediated pupil dilatation due to attenuation of the activity of the coeruleo-spinal pathway and also a reduction in noradrenergically-mediated inhibition of the EWN (See Fig. (**4**)). Post-synaptic receptors in the EWN are also stimulated by α_2_-adrenoceptor agonists resulting in an increase in noradrenergic inhibition of the EWN. The pupillary effects of clonidine indicate that in man the pre-synaptic effect of the drug is likely to predominate (see Table **1**). α_2_-Adrenoceptor agonists cause a reduction in pupil diameter (miosis); [184, 341, 379]. It has been shown that this effect is more pronounced in light than in darkness, consistent with attenuation of the noradrenergic inhibition of parasympathetic outflow to the iris [444]. In agreement with this observation, it has been shown that clonidine [39] and dexmedetomidine [238] enhance the amplitude of the light reflex response. Remarkably, µ opiate receptor agonists (eg. morphine) also cause miosis in man, likely to reflect the close association between α_2_-adrenoceptors and µ opiate receptors on LC neurones (see below, 3.1.1.6).

In contrast to clonidine, the α_2_-adrenoceptor antagonist yohimbine increases pupil diameter in human subjects [296, 341, 342]. Furthermore, like the effect of clonidine on the pupil, this effect was light-dependent, suggesting an increase in the noradrenergic inhibition of parasympathetic outflow to the iris.

##### 3.1.1.3 Blood Pressure

The LC exerts both direct and indirect effects on the activity of preganglionic sympathetic neurones in the IML. The direct effect is mediated *via* a descending excitatory noradrenergic pathway, whereas the indirect effect involves the modulation of the activity of other premotor sympathetic nuclei (RVLM, caudal raphe nuclei, A5 noradrenergic neurones, C1 adrenergic neurones) projecting to the IML [27, 422] (see also Section 2.3.2 in Part I). While the noradrenergic projections from the LC to the serotonergic neurones of the caudal raphe, the A5 noradrenergic and C1 adrenergic neurones are likely to be excitatory, the projection to the RVLM is inhibitory (see Fig. (**4**)). The RVLM itself exerts a sympathoexcitatory effect *via* a glutamatergic projection to the IML (see 2.3.2.1, Part I). Therefore, the direct noradrenergic projection to the IML and the indirect projection *via* the RVLM have opposing effects on blood pressure: the effect of the stimulation of preganglionic sympathetic neurones is counteracted by the removal of the sympathoexcitatory influence of the RVLM. α_2_-Adrenoceptor agonists have robust hypotensive effects due to the summation of their direct sympatholytic effect resulting from simulation of presynaptic autoreceptors and augmentation of the noradrenergic inhibition of the RVLM resulting from activation of post-synaptic α_2_-adrenoceptors in this nucleus (see Table **1**). The hypotensive effects of clonidine are well documented [15, 45, 126, 183, 185, 261, 296, 342, 379, 421, 486]. Furthermore, clonidine is marketed as an effective anti-hypertensive drug.

The role of the RVLM in mediating the hypotensive effect of α_2_-adrenoceptor agonists is well demonstrated [47, 163, 238]. Indeed, microinjection of clonidine directly into the RVLM results in hypotension and it is suggested that these neurones are tonically inhibited by α_2_-adrenoceptor activation [474]. The input from the LC is also believed to contribute to the baroreceptor reflex pathway outlined above through activation of these α_2_-adrenoceptors [154, 308], and this is supported by studies of α_2A_ knockout mice that demonstrate impaired baroreceptor reflex function [308]. Other α_2_-adrenoceptor agonists, such as dexmedetomidine also exert a pronounced depressor effect [135, 269]. The reduction in heart rate and blood pressure by dexmedetomidine has been found to result from a reduction in sympathetic activity [269] and an augmentation of parasympathetic activity [336], consistent with LC inactivation.

Again, yohimbine modifies blood pressure in a direction that is opposite to that produced by clonidine: pressor responses have been reported [148, 204, 342, 483], consistent with disinhibition of pre-synaptic autoreceptors in the LC and post-synaptic inhibitory receptors in the RVLM. The involvement of the RVLM in the hypertensive action of α_2_-adrenoceptor antagonists is highlighted by the observation that microinjection of methoxy-idazoxan into this area of the brainstem resulted in an increase in blood pressure [474].

It should be noted that α_2_-adrenoceptors also occur in peripheral blood vessels where they mediate a vasoconstrictor effect [77, 299]. The vasoconstrictor effect of clonidine, however, does not lead to an increase in systemic blood pressure since its effect on blood pressure is superseded by the central sympathoinhibitory effect of the drug. However, when the influence of central sympathoinhibition is disrupted due to lesions of the spinal cord, clonidine evokes an increase in blood pressure due to its peripheral vasoconstrictor effect [393].

##### 3.1.1.4 Salivation

Salivary glands are controlled mainly by a parasympathetic innervation, the preganglionic neurones in the salivatory nuclei being located in the brainstem (see 2.3.1.2, part I). The LC exerts an inhibitory influence on these neurones. Clonidine and other α_2_-adrenoceptor agonists lead to a substantial reduction in salivary output [15, 45, 184, 207, 261, 342, 379, 388, 421, 446, 486] consistent with the preferential activation of post-synaptic α_2_-adrenoceptors on salivatory neurones (see Table **1**). On the other hand, α_2_-adrenoceptor antagonists like yohimbine lead to hypersalivation by blocking inhibitory α_2_-adrenoceptors on salivatory neurones and thus attenuating noradrenergic inhibition of the activity of these neurones [204, 292, 342, 446]. It should be noted, however, that α_2_-adrenoceptor agonists may reduce and α_2_-adrenoceptor antagonists may enhance salivation not only by interacting with α_2_-adrenoceptors on preganglionic neurones in the salivatory nuclei but also with release inhibiting α_2_-adrenoceptors on the terminals of postganglionic cholinergic neurones in the salivary glands [292, 446]. 

##### 3.1.1.5 Temperature

There is evidence that the VLPO is intimately involved in temperature regulation [387] and also that this structure receives a dense noradrenergic innervation from the LC (see 2.2.2.1, Part I). Interestingly, the LC is involved in modulating the hypothalamic regulation of body temperature. It has been reported that the LC is involved in lipopolysaccharide-induced fever [362], which is attenuated by LC lesions [9]. Furthermore, LC lesions reduce brain temperature [142] and hypothermia induced by hypoxia [112]. 

Clonidine, probably due to a reduction in LC activity, leads to a fall in body temperature both in experimental animals [241, 366, 461, 511] and humans [15, 138]. The hypothermic effect of clonidine is antagonised by yohimbine [366, 461] and by high ambient temperature, leading to an increase in body temperature [15].

##### 3.1.1.6 α_2_-Adrenoceptor Associations

###### Opiate Receptors

μ-Opiate receptors occur on LC neurones (see 3.2 in Part I), and opiate peptides (enkephalins, dynorphins) are highly concentrated in the LC [120, 297, 513]. It has been shown that μ-opiate receptors are co-localised with α_2_-adrenoceptors in the LC and their activation results in cellular inhibition *via* a shared potassium channel [75, 121]; see also 3.2, Part I). Morphine, like clonidine, reduces LC neurone activity [395] and induces synchronous oscillatory discharges [518]. Therefore, it is not surprising that there is a considerable pharmacological overlap between the actions of α_2_-adrenoceptor and opiate receptor-selective agonists. Thus, it has been reported that chronic treatment with morphine results in up-regulation of α_2_-adrenoceptors in the brain [155]. 

Morphine, like clonidine, is highly sedative in man, and this effect is likely to be due to attenuation of the activity of the LC (see Fig. (**4**) and Table **1**). Indeed, microinjection of morphine directly into the LC has been found to reduce arousal, observed as an increase in SWS [131]. Other opiate receptor agonists have also been found to produce sedation (oxycodone [476], tramadol [243, 249], codeine [277]).

Morphine, like clonidine, has been observed to reduce pupil diameter in man [104, 179, 225, 479], and a ‘pinpoint pupil’ is a clinical hallmark of opiate addiction. This effect of morphine is consistent with a decrease in LC activity leading to the disinhibtion of the parasympathetic light reflex mechanism. Other opiates share this effect (oxycodone [476], tramadol [225, 512], codeine [225, 479]). The close association between α_2_-adrenoceptors and μ-opiate receptors in the pupil control system is highlighted by the observation that the α_2_-adrenoceptor antagonist yohimbine can antagonise the mydriatic effect (see 3.2.6, below) of morphine in the rat [224].

An important therapeutic implication of the pharmacological association between α_2_-adrenoceptor and μ-opiate receptor mediated mechanisms is the usefulness of the α_2_-adrenceptor agonists clonidine and lofexidine to alleviate the opiate withdrawal syndrome [143]. Indeed, it has been shown that the LC plays an important role in the processes underlying opiate withdrawal [20, 228, 236].

###### Imidazoline Receptors

Clonidine and some other α_2_-adrenoceptor agonists with an imidazoline structure may interact not only with α_2_-adrenoceptors but also with imidazoline receptors [46, 329]. Imidazoline receptors have been subdivided into three subtypes (I_1_, I_2_ and I_3_) [165]. I_1_-imidazoline receptors have been shown to occur in the RVLM, and it has been proposed that these receptors are the most important in mediating the cardiovascular effects of I_1_-receptor agonists, such as moxonidine. The co-localisation of α_2_-adrenoceptors and imidazoline receptors may be restricted to the RVLM since imidazoline receptors have not been identified in the LC [447] and there is evidence suggesting that they may also be absent from salivatory nuclei since moxonidine does not reduce salivation [156]. The depressor effect of non-selective drugs, such as clonidine, would reflect an action both at imidazoline receptors and α_2_-adrenoceptors in the RVLM [156] and at α_2_-adrenoceptors in the LC. On the other hand, selective (non-imidazoline) antihypertensive drugs would exert their depressor effect by stimulating α_2_-adrenoceptors in the LC. It should be noted, however, that the nature of imidazoline receptors as an entity has been questioned and the relationship to α_2_-adrenoceptors remains controversial [164, 229].

##### 3.1.1.7 Relationship between Pre-synaptic and Post-synaptic Effects

α_2_-Adrenoceptors and μ-opiate receptors occur both pre-synaptically in the LC and post-synaptically on target cells receiving a noradrenergic innervation from the LC (see Fig. (**4**)). The effect of agonists acting at these receptors will be determined by the relationship of the effects resulting from the activation of receptors at the pre-synaptic and post-synaptic sites. In the case of arousal and pupil diameter regulation, the action of the agonists at the two sites leads to opposing effects: activation of α_2_-adrenoceptors or μ-opiate receptors in the LC results in sedation and pupil constriction, whereas activation of the same receptors in the target cells (VLPO and EWN, respectively) leads to increase in alertness and pupil dilatation. Interestingly there is a remarkable species difference regarding the action of α_2_-adrenoceptor and μ-opiate receptor agonists on arousal and pupil diameter, and this species difference is likely to reflect the preponderance of the action of these drugs at either pre-synaptic or post-synaptic receptors (see Table **3**). Thus, while the α_2_-adrenoceptor and μ-opiate receptor agonists are uniformly sedative in man, dog and rabbit, they often have alerting effects, usually demonstrated as EEG activation, in cats, rats and mice. It should be noted; however, that sedative effects of α_2_-adrenoceptor agonists have also been demonstrated in cats [280], rats [305], and mice [167], and there is evidence that the activation of presynaptic α_2_-adrenoceptors in the LC is responsible for this effect [168, 305]. There is also a species difference regarding the pupillary effect of yohimbine: whilst in humans it produces mydriasis, in cats and rats it evokes miosis [177].

As pre- and post-synaptic receptors exist in all the species studied, it is likely that in man, dog and rabbit where the presynaptic effect predominates this reflects the generally higher sensitivity of pre-synaptic autoreceptors compared to post-synaptic receptors, leading to preferential activation of pre-synaptic receptors at lower dosage levels of α_2_-adrenoceptor and μ opiate receptor agonist. The presence of post-synaptic receptor activation in these species is highlighted by the observation that pupil dilatation has been observed in two human patients with high plasma morphine concentrations [397], and clonidine, while it is highly sedative in dogs at smaller doses, evokes behavioural stimulation at higher dosage levels [180]. However, in the cat, rat and the mouse where the post-synaptic effect usually predominates, it is likely that the pre-synaptic effect is masked by the consequences of post-synaptic receptor activation.

#### 3.1.2 Drugs Interacting with Reuptake

Reuptake of released noradrenaline into the nerve terminal is the principle way of terminating the synaptic action of the transmitter. Reuptake involves the operation of an active membrane pump (noradrenaline transporter; [10, 53, 274]. Inhibitors of the transporter result in attenuation of reuptake leading to potentiation of the effect of the transmitter on the post-synaptic cell. Inhibitors of noradrenaline reuptake are expected to enhance the post-synaptic effects of LC activation in each target area. We shall consider the same four functions (i.e., arousal, pupil control, blood pressure control, salivation) as discussed in relation to modulation by autoreceptors (see 3.2 above and Fig. (**4**)).

The noradrenaline transporter has been indentified on noradrenergic neurones in the LC using the highly selective noradrenaline uptake inhibitor nisoxetine [405, 456]. Noradrenaline uptake inhibitors can influence the removal of noradrenaline released from dendrites in the LC leading to increased activation of inhibitory autoreceptors *via* the increased noradrenaline concentration resulting in a reduction in LC activity [145]. This effect, however, is likely to be superseded by the effect of noradrenaline uptake inhibition at the nerve terminals since while both amphetamine [7, 86] and cocaine [89, 348, 349, 350] reduce the activity of the LC, they also increase the concentration of noradrenaline in the synaptic gap at the target areas of noradrenergic terminals [86] leading to increases in LC-mediated functions such as arousal and pupil diameter (see below). 

##### 3.1.2.1 Arousal

Inhibition of noradrenaline uptake at the excitatory noradrenergic synapses in the cerebral cortex and in wakefulness-promoting nuclei, together with a similar action at inhibitory noradrenergic synapses in the VLPO, is expected to lead to an increase in arousal. Noradrenaline uptake inhibition in general has been associated with an alerting effect. Thus, it has been shown that the noradrenaline uptake inhibitors indeloxazine [403] and S33005 [62] increase alertness in experimental animals.

Although the psychostimulant actions of amphetamine and cocaine are usually attributed to the blockade of the reuptake of dopamine, these drugs are also potent inhibitors of noradrenaline reuptake [495]. These drugs produce robust increases in arousal (*amphetamine* [157, 171]; *cocaine* [492]). The wakefulness-promoting drug modafinil, which is not classified as a psychostimulant, also increases alertness [184, 187, 380]; see 3.1, above) and inhibits the reuptake of both dopamine and noradrenaline [262], and the site at which noradrenaline uptake inhibition may be most relevant has been identified as the inhibitory noradrenergic synapse on VLPO neurones [128].

A number of antidepressants have been developed whose only or principal action is the inhibition of noradrenaline reuptake (e.g., reboxetine, venlafaxine, atomoxetine, duloxetine). Some of these drugs also have, albeit relatively weak, alerting effects. Reboxetine has been claimed to be an “activating” antidepressant improving social activity in depressed patients [98] and both reboxetine and atomoxetine are used as alternatives to psychostimulants in the treatment of attention deficit hyperactivity disorder [517]. Reboxetine has also been shown to increase arousal and suppress REM sleep in rats [227]. Phentermine, an anorectic agent used in the treatment of obesity, also inhibits noradrenaline and dopamine reuptake and has been shown to increase wakefulness in rats [371].

##### 3.1.2.2 Pupil Control

Inhibition of noradrenaline uptake is expected to potentiate the noradrenergic excitation of preganglionic sympathetic neurones in the IML, the stimulant effect of noradrenline at the neuroeffector junction in the iris, and the noradrenergic inhibition of the EWN. Indeed, both amphetamine [151, 157, 176, 351] and cocaine [202, 351, 497] have been demonstrated to increase pupil diameter consistent with such an action. Furthermore, there is evidence that amphetamine-induced pupil dilatation is related to the inhibition of parasympathetic output [224], consistent with an increase in the noradrenergic inhibition of the EWN.

It has been shown that both reboxetine [445] and venlafaxine [41] cause an increase in pupil diameter and inhibit the light reflex response. Although the increase in pupil diameter may simply reflect noradrenaline reuptake in the iris, leading to the enhancement of sympathetically-mediated pupil dilatation, the inhibition of the light reflex demonstrates the involvement of an action at the noradrenergic synapse on EWN neurones. Consistent with these findings, the predominantly noradrenergic tricyclic antidepressant desipramine also increases pupil diameter [406] and inhibits the light reflex [459]; however, these effects may have been contaminated by an interaction of the drug with muscarinic cholinoceptors.

##### 3.1.2.3 Blood Pressure

Noradrenaline uptake inhibition is expected to lead to augmentation of blood pressure [279], partly due to potentiation of the central sympathoexcitatory effect of the noradrenergic input to preganglionic sympathetic neurones and partly to potentiation of noradrenergically-mediated vasoconstriction in the periphery. This pressor effect is expected to be attenuated by potentiation of the noradrenergic inhibition of the RVLM. This attenuation is likely to be significant since noradrenaline reuptake inhibitors result in only moderate increases in blood pressure. 

Administration of both amphetamine [59, 157, 264, 307] and cocaine [178, 284, 307] is associated with increases in blood pressure. Reboxetine [445], venlafaxine [2] and duloxetine [458] have all been reported to cause modest increases in blood pressure.

##### 3.1.2.4 Salivation

Potentiation of the noradrenergic inhibition of the salivatory nuclei, as a result of noradrenaline reuptake inhibition, is expected to result in a reduction in salivary output. Indeed, both reboxetine [445] and venlafaxine [2] have been shown to reduce salivation. These observations show that a reduction in salivation by an antidepressant is not necessarily due to muscarinic cholinoceptor blockade in the salivary glands, as generally assumed, but may reflect the interaction of the drug with central noradrenergic mechanisms [445].

#### 3.1.3 Drugs Interacting with Storage

Noradrenaline is stored in synaptic vesicles in the nerve terminal, and its accumulation in the storage vesicles is *via* an active membrane pump (vesicular monoamine transporter 2, VMAT2; [516]). VMAT2 is not specific for noradrenaline, and is responsible for transporting also dopamine and serotonin into storage vesicles in the appropriate nerve terminals. VMAT2 is inhibited by some drugs such as reserpine and tetrabenazine, leading to the initial release of the monoamine followed by depletion of the stores, resulting in reduction/cessation of release [107]. While dopaminergic neurones show preferential sensitivity to depletion by tetrabenazine [449]; [132], reserpine leads to near equal depletion of all three monoamines [406]. The depletion of noradrenaline from central noradrenergic neurones leads to the “switching off” of the LC system, whereas depletion of noradrenaline from peripheral noradrenergic neurones results in a marked sympatholytic action [436]. 

Early work with the depleting agents carried out in the 1960s has described the consequences of noradrenaline depletion on functions mediated by the LC system, and more recent information relating to the functional neuroanatomy of the system (see Fig. (**4**)) enables us to interpret these effects. Thus, administration of reserpine leads to sedation [436], which may be related to reductions in the excitatory influence of the LC on the cerebral cortex and other wakefulness-promoting nuclei and of the noradrenergic inhibition of the sleep-promoting VLPO. The “switching off” of the noradrenergic inhibition of the EWN is expected to lead to increased activity of this preganglionic parasympathetic nucleus. Indeed, it has been shown that reserpine causes miosis, which is more pronounced at ambient illumination than in darkness, and potentiates the light reflex [43]. Reserpine also leads to marked hypotension, presumably due to the combination of its central and peripheral sympatholytic effects [436]. Reserpine-induced noradrenaline depletion has also been demonstrated to be associated with an increase in salivary output [43], which is likely to be due to the removal of the tonic noradrenergic inhibition of salivatory nuclei.

### 3.2 Drugs Indirectly Modifying LC Activity

As reviewed in Part I, the LC receives afferent inputs from a large number of sources. In this section we wish to restrict ourselves to reviewing modifications in LC activity by afferent inputs arising from the sleep-arousal network (see Fig. (**1**)). Stimulant and sedative drugs, apart from directly influencing LC activity (see 3.1, above), often exert an indirect effect on the LC *via* acting at different sites in the sleep-arousal network. 

#### 3.2.1 Stimulant Drugs

##### 3.2.1.1 Adenosine Receptor Antagonists

Adenosine accumulates during wakefulness and plays a role in the initiation of sleep, *via* activation of A_1_ and A_2_ adenosine receptors [117, 466]. The sleep-inducing effect of adenosine is mediated partly *via* inhibition of wakefulness-promoting nuclei [287] and partly *via* stimulation of sleep-promoting nuclei [129, 190], which are known to project to the LC (see Fig. (**2**)).

*Caffeine* is an adenosine receptor antagonist which is widely available and used extensively in the general population as a wakefulness-enhancing compound. The drug produces robust increases in arousal [25, 54, 213, 335, 488, 522] and has been shown to enhance LC activity [28, 100]. Administration of caffeine also produces increases in heart rate [194], blood pressure [194, 315, 464] and temperature [229], consistent with activation of the sympathetic nervous system. Interestingly, caffeine antagonises the sedative and autonomic effects of clonidine [417], a drug known to reduce LC activity (see 3.1, above). 

##### 3.2.1.2 Drugs Interacting with the Mesocoerulear Pathway

The dopaminergic system plays an important role in the maintenance of arousal [258] and psychostimulants such as amphetamine and cocaine are potent activators of this system by inhibiting the dopamine transporter and facilitating dopamine release [48, 368]. The alerting effect of dopaminergic drugs may partly be mediated *via* the LC since it has been shown that this nucleus receives excitatory inputs both from the VTA (mesocoerulear pathway) and vPAG (see 3.1.4.1 and 3.1.4.4, Part I).

*Modafinil* is a novel wakefulness-promoting drug used in the treatment of EDS in narcolepsy [468, 469] and it has been shown to alleviate the EDS associated with a number of other conditions (PD [310]; idiopathic hypersomnia [199]; night shift sleep disorder [480]; obstructive sleep apnoea [325]; multiple sclerosis [358]; myotonic dystrophy [260]; depression [96]; schizophrenia [370]; sleep deprivation [346]; and drug-induced sedation [485]. Modafinil has been found to enhance the activity of the dopaminergic system by increasing extracellular levels of dopamine [102, 298, 499] possibly by an action at the dopamine transporter [262, 310]. Consequently, it has been proposed that modafinil may potentiate LC activity *via* the enhancement of dopaminergic activity within the mesocoerulear pathway originating in the VTA [377, 383, 384]. It should be noted however, that modafinil failed to modify LC activity in anaesthetised animals [7] where the baseline firing rate of the neurones is very low. It has been suggested that this study should be repeated in awake animals where the LC fires at a higher rate and may be more sensitive to modafinil [383]. 

Modafinil’s pharmacological effects are consistent with those associated with increased LC activity (see 3.1 and Fig. (**4**), above). Modafinil has been found to increase arousal in healthy volunteers [184, 187, 379, 488], together with signs of sympathetic activation, including increases in pupil diameter [184, 187, 379], heart rate and blood pressure [187, 264, 377, 379], and body temperature [283, 346]. Interestingly, there is little evidence of a concomitant decrease in parasympathetic activity, since light reflex amplitude and salivary output are not affected by the drug [184, 187, 377, 379]. This observation suggests that the mesocoerulear pathway may preferentially activate the pre-sympathetic neurones in the LC (see 5 and Fig. (**6**), below).

*Amisulpride* is an antipsychotic with an antagonistic action at D_2_ dopamine receptors. D_2_ dopamine receptors have been shown to occur both pre-synaptically on dopaminergic neurones (autoreceptors) and post-synaptically: the pre-synaptic receptors inhibit the firing of the neurone [21], whereas the post-synaptic receptors may mediate either excitatory or inhibitory effects on the post-synaptic cell. The pre-synaptic receptors are generally more sensitive to both agonists and antagonists than the post-synaptic receptors, and amisulpride has a preferential action at the pre-synaptic autoreceptors [291, 337]. In this respect amisulpride differs from most other antipsycotics which although block D_2_ dopamine receptors [222, 432], do not show any selectivity for the pre-synaptic site [222, 291, 337]. In the case of the mesocoerulear pathway, the preferential blockade of inhibitory pre-synaptic receptors would increase the activity of the VTA neurones. As the dopamine released onto LC neurones is likely to interact with excitatory D_2_ dopamine receptors, the activity of the LC would increase leading to increase in alertness. Indeed, amisulpride has been shown to possess some mild alerting effects [331], in contrast to antipsychotics without pre-synaptic D_2_ dopamine receptor selectivity which have no alerting effect and are usually sedative in action [222].

#### 3.2.2 Sedative Drugs

##### 3.2.2.1 GABA Receptors

GABA is the major inhibitory neurotransmitter in the brain, and it exerts its action by interacting with three types of receptor (GABA_A_, GABA_B_, and GABA_C_). A major class of sedative drug, the *benzodiazepines*, interact with specific benzodiazepine receptors which are closely associated with the GABA_A_ receptor. Activation of benzodiazepine receptors leads to potentiation of the inhibitory actions of GABA [289, 347]. As most sleep-promoting neurones utilise GABA as their neurotransmitter, benzodiazepines act by enhancing the influence of sleep-promoting systems.

It has been shown that benzodiazepines reduce the firing rate of LC neurones [239, 382, 410], and it has been proposed that the LC may play an important role in mediating the sedative effects of these drugs [240]. However, the reduction in LC activity after administration of benzodiazepines is unlikely to be due to potentiation of the inhibitory action of GABA on LC neurones since the GABA_A_ receptors on these neurones are not sensitive to benzodiazepines [71]. Therefore, the suppression of LC activity by benzodiazepines is likely to reflect enhancement of GABAergic inhibition at other sites in the sleep-arousal network, e.g., in the cerebral cortex and TMN, structures with excitatory inputs to the LC (see 3.1.1 and 3.1.3.4, Part I). The benzodiazepine diazepam has been found to attenuate the stress-induced increases in noradrenaline turnover at sites such as the hypothalamus, amygdala, hippocampus and cerebral cortex [197], all areas which receive noradrenergic innervation originating in the LC. Interestingly, chronic diazepam administration enhances LC activity [338] and this over-activity may form part of the circuitry underlying benzodiazepine dependence and withdrawal.

It is generally recognised that there is a close relationship between the level of arousal and pupil diameter: sedation is known to be associated with miosis, as observed for the α_2_-adrenoceptor agonists and opiates (see 3.1, above), and pupil diameter is often used by anaesthetists to monitor the depth of anaesthesia (p 779-781 in ref. [254]). The anatomical substrate of this relationship is likely to be the LC (see 1.3, above). Therefore, it is surprising that the benzodiazepines, while highly sedative, do not cause any change in pupil diameter [186, 189], although they reduce LC activity (see above) and induce fluctuations in the firing rate of LC neurones as reflected in the enhancement of pupillary fatigue waves [189]. These observations suggest that the benzodiazepines, apart from reducing LC activity, may also have a direct effect on the pupil control mechanism which masks the miosis expected from the reduction in LC activity. This mechanism has been suggested to be enhanced sympathetically-mediated mydriasis resulting from reduced inhibition of the IML due to potentiation of GABA-mediated inhibition of descending inhibitory bulbospinal pathways and inhibitory interneurones [189].

##### 3.2.2.2 Mesocoerulear Pathway

As discussed above, LC activity is influenced by a dopaminergic input from the VTA, the mesocoerulear pathway, operating *via* D_2_ dopamine receptors (see 3.2.1.2, above). Pre-synaptic autoreceptors play an important role in modulating the activity of VTA neurones, their activation leading to a decrease and their inhibition to an increase in neuronal activity. D_2_ dopamine receptor agonists, with a preferential affinity for the autoreceptor, lead to attenuation of the dopaminergic facilitation of LC activity resulting in lowering of the level of arousal [217, 373, 377, 378, 380]. Indeed, it has been shown that D_2_ dopamine receptor agonists used in the treatment of PD have profound sedative effects (for example, pramipexole [161]; ropinirole [116]; pergolide [463]; bromocriptine and cabergoline [333]; piribedil [452]: apomorphine [244]). These drugs are likely to exert their therapeutic effects *via* the activation of dopamine receptors within the striatum [217, 373].

*Pramipexole* is a dopamine D_2_ receptor agonist which has been shown to possess robust sedative effects in healthy volunteers [387, 388, 390]. However, paradoxically pramipexole-induced sedation was associated with an increase rather than a decrease in pupil diameter as would be expected on the basis of reduced LC activity. The increase in pupil diameter was accompanied by a reduction in light reflex amplitude, indicating a reduction in the parasympathetic outflow to the iris. It was proposed that the mydriatic effect of pramipexole might reflect the operation of a putative excitatory pathway from the VTA to the EWN (“meso-pupillomotor” pathway) whose “switching off” by autoreceptor stimulation would be responsible for the attenuation of parasympathetic activity [387, 388, 390]. Interestingly, this proposal is supported by the effect of the D_2_ autoreceptor antagonist amisulpride, which caused miosis and an increase in light reflex amplitude [388].

## 4 AGE-DEPENDENT ALTERATIONS OF LOCUS COERULEUS ACTIVITY

Neurone density within the LC decreases with age due to a progressive loss of noradrenergic neurones, both in animals [83, 246, 434] and humans [65, 265, 460, 477]. This neurone loss is uniformly diffuse across the LC, without preferential reduction in any restricted area [270]. In addition, the remaining neurones within the LC show shrinkage of the perikarya and a subsequent decrease in cell size [65, 83]. Ageing cells within the LC also exhibit mitochondrial and ribosomal alterations and these changes have been suggested to produce disruptions in the absorption of nutrients, protein synthesis, energy supply, transport of materials and message exchange [83]. The age-dependent decline in LC activity may be accentuated *via* an indirect mechanism. The ageing brain also shows a reduction in orexin B-immunoreactive axons within the LC accompanied by a decline in tyrosine hydroxylase mRNA in the LC, a marker of noradrenergic activity [105]. 

It has been observed that the number of LC neurones projecting to areas such as the frontal cortex and the hippocampus declines with age, but that a certain degree of axonal branching occurs to maintain noradrenaline levels at target areas [196, 197, 403]. However, despite this branching, the loss of noradrenergic LC axons innervating the frontal cortex has been found to result in fewer synapses [198], and changes in the electrophysiological properties of the remaining LC axon terminals have been observed [404]. Decreased binding in the LC of nisoxetine, a noradrenaline uptake inhibitor, has been observed in aged humans [456] and this decrease is likely to be related to the loss of LC neurones, and thus a reduction in noradrenaline transporters at nerve terminals. Similarly, in an aged rat nisoxetine-sensitive noradrenaline uptake inhibition was attenuated, again representing a decline in noradrenergic transporters at axon terminals [405]. In addition to a loss of transporters, α_2_-adrenoceptor responsiveness in the LC is reduced with ageing. In aged rats microinjections of clonidine and yohimbine into the LC, where these drugs have a localised effect on presynaptic inhibitory autoreceptors, do not modify alertness level, in contrast to the sedative and wakefulness-promoting effects, respectively, observed in younger rats [99, 312, 313]. Thus, these various disruptions in the functioning of the LC with age result in an overall deficiency in noradrenergic neurotransmission [34]. 

Memory impairment in old age has been related to the loss of LC function with ageing [272]. For example, reductions in cortical noradrenaline transmission with age have been associated with deficits in spatial learning and memory [78] and a significant correlation has been observed between the extent of neurone loss in the LC with age and the degree of memory impairment on an inhibitory avoidance task in mice [246].

This influence of ageing on LC activity can be observed in changes in pupillary function. Resting pupil diameter is reduced with age [37, 352, 416, 431], with pupil diameters of 1 mm or less becoming more prevalent [423], and this is consistent with a deficit in the sympathetic nervous system resulting from reduced LC activity. A decline in the sympathetic outflow to the iris in old age is further demonstrated by reductions in the amplitude and velocity of the darkness reflex response and prolongation of the recovery time of the light reflex response [37]. However, the parameters of the light reflex response may also be altered in old age [37, 352, 390, 431], suggesting that the aging process may affect both the sympathetic and parasympathetic controls of the pupil. The role of the LC in the age-related sympathetic deficit in pupillary control is highlighted by the fact that there is a parallelism between the monotonic decline in pupil size [416] and cell numbers in the LC [265] with age. The age-related decline in LC neurone numbers is likely to be accelerated in AD, resulting in accentuation of the sympathetic deficits of pupil control present in elderly people (see 5.2, below).

## 5 PATHOLOGICAL ALTERATIONS OF LOCUS COERULEUS ACTIVITY

Alongside the physiological and pharmacological manipulations that can produce changes in the activity of the LC, pathological changes associated with various diseases can alter LC neurone functioning. Pathological changes in the LC have been noted in a number of neurodegenerative diseases, including PD [514], AD [44], Huntington’s disease [521], progressive supranuclear palsy [266], Lewy body disease [435], Down’s syndrome [271], Pick’s disease [13], and amyotrophic lateral schlerosis [318]. In this review we focus our attention on the most common of these: PD and AD. In addition, the possible contribution of disruption to LC neurone function in brainstem coma is discussed.

### 5.1 Parkinson’s Disease

PD is a progressive neurodegenerative disorder associated with motor deficits that include muscular rigidity, bradykinesia, resting tremor, postural instability and hypokinesia. These deficits have been largely related to the degeneration of dopaminergic neurones in the pars compacta of the substantia nigra that project to the striatum, leading to a reduction in dopamine content within the striatum. However, there is evidence that the noradrenergic neurones of the LC are also important in this disorder [63, 136, 272]. A significant loss of LC neurones has been observed post-mortem in PD [32, 133, 332, 356, 472] and also indicated by a reduction in the neuromelanin signal localised to the LC using MRI in living patients ([386]; see 4.5, below). Indeed, neurone loss from the LC has been found to be more extensive than that from the substantia nigra [514], with Lewy body formation commencing in a number of areas including the LC before any pathology occurs in the substantia nigra [49, 97]. 

In addition to a loss of LC neurones, PD is also associated with morphological changes to the surviving neurones in the LC, including changes in the size and shape of both pre- and postsynaptic components of synapses, polymorphisms of the synaptic vesicles, changes in the morphology of the mitochondria, swelling of the cell bodies, inclusion of Lewy bodies, and shortening and thinning of the dendrites with absent or reduced arborisations [23, 64, 332].

It is known that the LC sends an excitatory noradrenergic projection to the striatum-projecting neurones of the substantia nigra [79, 146], activating the dopaminergic neurones *via* activation of α_1_-adrenoceptors [146]. Interestingly, lesions of the LC result in an increase in the loss of dopaminergic neurones from the substantia nigra induced by the neurotoxin 6-OHDA [35, 425], indicating that the noradrenergic neurones from the LC have a neuroprotective effect on the dopaminergic neurones of the substantia nigra. In addition, in monkeys with MPTP induced PD, lesions of the LC impair the recovery of the motor deficits that usually occurs at 9 weeks post-treatment [276], supporting the involvement of the LC in the progression of PD. It, therefore, seems likely that the noradrenergic neurones of the LC have both neuromodulatory effects on dopaminergic neurones of the substantia nigra, important for maintaining or facilitating the nigrostriatal pathway, and neuroprotective effects on these dopaminergic neurones, resulting in neurodegeneration when the LC is compromised and contributing to the development of PD [272, 424]. 

Sleepiness in PD has been found to be related to the pathology of the disease alongside any sedation induced by pharmacological treatment [14] and it is likely that this sleepiness results from the reduction in LC activity that occurs as a result of the neurone loss described above. In addition, the degree of LC neurone loss has been associated with the incidence of dementia in PD [520] and this association may be related to the loss of LC neurones involved in the circuitry underlying executive functioning (see Part I). In support of this suggestion, noradrenaline levels in the LC have been found to be reduced post-mortem in PD patients who also suffered from dementia compared to PD patients who had no clinical signs of dementia [60]. Alongside the sleepiness and dementia discussed above, depression and anxiety in PD may also be associated with the loss of LC neurones, resulting from a reduction in noradrenergic innervation of the limbic system [367].

Pharmacological studies suggest that α_2_-adrenoceptor antagonists could be used therapeutically in an attempt to increase LC neurone activity in PD. For example, in a primate model of PD administration of the α_2_-adrenoceptor antagonist idazoxan improved motor abnormalities [33]. This possibility requires further investigation.

### 5.2 Alzheimer’s Disease

AD is a progressive neurodegenerative disorder associated with a decline in cognitive functions including memory, language, attention, judgement and executive function (for example, planning and organisation). These deficits have primarily been related to dysfunction within the cholinergic system, particularly in the nucleus basalis. However, there is considerable evidence that dysfunction within the LC is also important in this disorder, since a significant loss of noradrenergic LC neurones can be observed post-mortem [58, 63, 64, 125, 133, 201, 259, 267, 272, 275, 433, 448, 472, 503]. This loss of LC neurones is greater than that observed in the case of normal ageing ([460]; see below) and interestingly there is significantly more neurone loss from the LC than from the cholinergic neurones in the nucleus basalis [267, 514]. It has been proposed that LC neurone degeneration may contribute to the development and progression of AD, since in amyloid-containing amyloid precursor protein 23 (APP23) transgenic mice, a model of AD pathology, LC lesions augmented amyloid plaque deposits in the projection areas of the LC and increased memory deficits compared to non-transgenic mice who also received LC lesions [172]. The root cause of the loss of neurones from the LC in AD is unclear, but may be associated with the formation of neurofibrillary tangles, since neurofibrillary tangles have been identified within the LC [58, 503]. In addition, increased levels of the neurotoxic monoamine oxidase A metabolite of noradrenaline, 3,4-dihydroxyphenylglycolaldehyde, has been observed in the LC in AD and may contribute to this neurone loss [57]. It appears, however, that the LC neurone loss is not related to the over-expression of beta-amyloid protein [134].

Alongside the reduction in LC neurone density, mean brain noradrenaline concentration is lower in AD sufferers than in healthy controls [181] and cortical noradrenaline levels have been found to be reduced [201, 275]. In contrast, noradrenaline levels measured from the cerebro-spinal fluid are significantly higher in AD than in controls [110], and this may result from an increase in activity of some surviving neurones in order to compensate for the extensive neurodegeneration [181, 448]. Pathological changes are observable within these surviving LC neurones, however, where cell somata are reported as swollen and misshapen and dendrites are shorter and less branching [64].

The mechanism by which LC neurone loss contributes to AD development is unclear. In non-disease states noradrenaline from the LC may directly influence cell bodies of the nucleus basalis to increase acetylcholine release [272, 415, 509] and may modulate cortical cholinergic transmission *via* activation of α_2_-adrenoceptors on cholinergic terminals of cortically-projecting nucleus basalis neurones, again to increase acetylcholine release [170]. Therefore, a reduction in noradrenaline release from the LC in AD would result in a reduction in cholinergic neurotransmission. In addition, a loss of cholinergic innervation of the LC has been observed in AD [433] and this may contribute to the reduced LC functioning in AD discussed above.

As noradrenaline depletion studies using a neurotoxin to lesion the LC have found deficits in attention, learning and memory [272], neuronal loss in the LC may contribute to the cognitive decline in AD. It has been reported that as pathological changes in the LC occur early in AD, the early cognitive changes in the disorder may be related to LC degeneration [150]. LC degeneration is progressive during the course of the illness and there is a positive correlation between the duration of illness and the magnitude of LC neurone loss [133]. Furthermore, there are also positive correlations between the magnitude of LC neurone loss and dementia severity [44] and between the reduction in noradrenaline concentration in the cerebral cortex and cognitive impairment [275]. Interestingly, patients with a history of depression had significantly lower neurone numbers within the LC [125]. The LC may thus be a possible therapeutic target to alleviate the cognitive deficits in AD. It has been reported that transcutaneous electrical nerve stimulation (TENS), using the same stimulus parameters which have been shown to increase LC activity in experimental animals, results in improvements of both cognitive functions and behaviour in patients suffering from AD [389]. This observation opens the way to consider retrograde vagus nerve stimulation as a possible indication for AD, and indeed preliminary reports indicate that this procedure may have beneficial effects on cognitive functions in patients suffering from AD [285, 414]. The electrical stimulation of the left vagus nerve is well established as a therapeutic procedure both in epilepsy [149] and treatment-resistant depression [301]. Interestingly, it has been shown that vagus nerve stimulation leads to increases in the firing rates of noradrenergic neurones in the LC and serotonergic neurones in the dorsal raphe nucleus [103].

It may be expected that a reduction in LC activity, which is likely to occur from the earliest stages of AD, would be reflected in deficits in a number of LC-mediated functions. Indeed, there is evidence of a sympathetic deficit in AD patients, affecting sweat gland activity and pupillary function. A reduction in impulse traffic in sympathetic fibres innervating eccrine sweat glands results in hyposensitivity of the glands to intradermally injected carbachol ([443], see also 2.2.2), and it has been reported that patients suffering AD show reduced responsiveness of their sweat glands to carbachol, compared to age- and sex-matched control subjects [235]. It has also been reported that patients suffering from mild/moderate AD have reduced pupil diameters and attenuated darkness reflex responses in comparison to age matched healthy controls, indicating reduced sympathetic function, consistent with a reduction in LC activity [353].

Patients with AD may show enhanced sensitivity of their pupils to the mydriatic effect of topically applied tropicamide, a muscarinic cholinoceptor antagonist [391]. Although this observation has been related to degenerative changes in the EWN seen in AD [392], it is more likely to reflect the loss of LC activity. It has recently been found that administration of clonidine, a drug known to “switch off” LC activity (see 3.1.1), results in augmentation of tropicamide-evoked mydriasis in healthy volunteers [188]. This observation also suggests that administration of a single dose of clonidine is likely to enhance the sensitivity of the “tropicamide” eye-drop test in AD, providing possible means for the early detection of developing brain degeneration [188].

Reduction in LC activity in AD is also expected to be reflected in the level of alertness of AD patients. Indeed, it has been reported that patients suffering from AD have reduced flicker fusion frequency thresholds compared to age-matched healthy controls [87, 88], indicating a decline in the level of arousal, consistent with a reduction in LC activity in AD.

It has been reported that both saccadic [118, 183, 184, 191] and smooth pursuit [119, 191, 234, 510] eye movements are impaired in AD. Eye movements are controlled by an intricate neural network involving brainstem nuclei and the cerebral cortex and degenerative changes in several sites in this network may explain the eye movement deficiency reported in AD [372]. However, it is of interest that a reduction in LC activity may also contribute to the eye movement disorders. The LC sends an excitatory output to the oculomotor nuclear complex (see 2.3.5.4, Part I), and there is pharmacological evidence that drugs modifying LC activity lead to alterations in eye movements. Thus, α_2_-adrenoceptor agonists (dexmedetomidine, clonidine), which “switch off” the LC, reduce peak saccadic velocity in healthy volunteers [1, 139, 154], whilst α_2_-adrenoceptor antagonists (ethoxyidazoxan), which potentiate LC activity, increase peak saccadic velocity in healthy volunteers [81]. Furthermore, sedative drugs in general reduce peak saccadic velocity [24, 473], consistent with a reduction in LC activity evoked by these drugs. 

### 5.3 Brainstem Coma

The presence of coma following brainstem stroke has been linked to lesions of nuclei located in the pons, in particular with the LC [330]. In this study, patients with brainstem damage without coma had no damage, or very little unilateral damage, to these areas in the tegmentum including the LC. Thus, lesions in the upper pons which include the LC are implicated in the causation of coma in humans, consistent with the wakefulness-promoting functions of this nucleus.

### 5.4 Imaging of Neuromelanin in Humans

The LC and the substantia nigra are visible to the naked eye when the brain is dissected since both nuclei contain a pigment, neuromelanin [515, 519]. It has recently become possible to image the substantia nigra [386] and the LC [386, 399, 400] see Fig. (**5**) using neuromelanin-magnetic resonance imaging. The contrast ratio between the neuromelanin-containing nuclei and the surrounding brain tissue is used as an index of neuromelanin concentration. Preliminary reports indicate that in PD the neuromelanin content is reduced both in the substantia nigra and the LC compared to healthy controls [386] and the neuromelanin content in the LC shows an inverted-U relationship with age, highest levels being observable in middle aged individuals [399]. Furthermore, a reduction in neuromelanin signal in the LC has been reported in patients suffering from depression [400]. These encouraging reports should be followed up by further work aimed at clarifying the relationship between neuromelanin content and cell numbers in the LC, since while the number of LC neurones has been reported to decline with age and the development of neurodegenerative diseases (see above), the neuromelanin concentration of pigmented nuclei seems to increase with the advancement of age [515]. Finally, the most important question is how neuromelanin content can be related to LC activity.

## CONCLUSIONS

It is apparent that manipulations of a physiological, pharmacological or pathological nature produce consistent alterations in LC activity, affecting both arousal state and autonomic function. Clearly, the LC is a major nucleus in the central regulation of these functions and reliable outcomes from changes in LC activity can be observed, especially on pupillary measures. In general, the physiological manipulations discussed above (anxiety, noxious/painful stimulation, extreme ambient temperature) increase LC activity and thus result in heightened arousal and changes in autonomic function consistent with sympathetic activation (for example, pupil dilatation). Similarly, administration of drugs such as stimulants, α_2_-adrenoceptor antagonists and noradrenaline uptake inhibitors increase the influence of the LC on areas receiving noradrenergic innervation. In contrast, administration of sedatives and α_2_-adrenoceptor agonists reduce LC activity and result in a decrease in arousal and produce sympatholytic effects (for example, pupil constriction). Likewise, LC neurone loss in aging, PD, AD, and brainstem coma results in decreases in arousal and a reduction in activity of the sympathetic nervous system. It is of interest to note that the pathological changes within the LC that occur in PD and AD are similar in nature and thus these disorders may form two poles of a spectrum of neurodegenerative disease related to loss of LC neurones [272, 339]. Overall it is clear that the LC is centrally involved in controlling the regulation of arousal and autonomic function, and thus any manipulation (physiological, pharmacological or pathological) which alters LC activity will have consequences on aspects of this control. 

The pattern of the consequences of LC activation by different variables suggests that there may be separate populations of LC neurones associated with sympatho-excitatory and parasympathetic-inhibitory effects (Fig. (**6**)). Thus, noxious stimulation (see 2.1, above) and enhancement of the activity of the mesocoerulear pathway by the wakefulness-promoting drug modafinil (see 3.2.1, above) result in the relatively selective activation of pre-sympathetic LC neurones since these variables increase pupil diameter without any inhibition of the light reflex response. Indeed, modafinil has no influence on salivary output, a parasympathetically-mediated function. On the other hand anxiety (see 2.2, above), probably *via* activation of the amygdala, leads to preferential activation of pre-parasympathetic LC neurones leading to inhibition of the light reflex and reduction in salivation. 

In conclusion, recent developments in the functional neuroanatomy of the central noradrenergic system have enabled the rational interpretation of the effects of a number of centrally-acting drugs. Conversely, many drugs have proved to be useful tools in the dissection of the complex neural network regulating arousal and autonomic activity. This fruitful relationship between functional neuroanatomy and pharmacological probes is likely to grow further with the development of neuroanatomical research tools of higher resolution and drugs with increasing selectivity and specificity.

## Figures and Tables

**Fig. (1):**
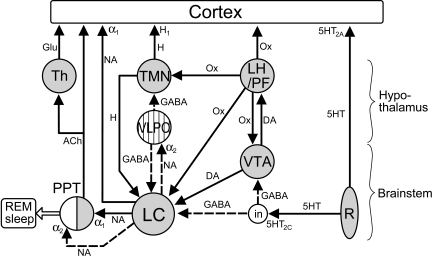
Schematic diagram of the connections within the arousal-controlling neuronal network. *Wakefulness-promoting nuclei* (grey): TMN, tuberomamillary nucleus; LH/PF, lateral hypothalamic/perifornical area; Th, thalamus; LC, locus coeruleus; VTA, ventral tegmental area; PPT, pedunculopontine tegmental nucleus; R, raphe nuclei. *Sleep-promoting nucleus* (hatched): VLPO, ventrolateral preoptic nucleus. GABAergic interneurones, in (white). *Neurotransmitters*: ACh, acetylcholine; NA, noradrenaline; H, histamine; Ox, orexin; GABA, γ-aminobutyric acid; DA, dopamine; 5HT, 5-hydroxytryptamine; Glu, glutamate. *Receptors*: α_1_, excitatory α_1_-adrenoceptors; α_2_, inhibitory α_2_-adrenoceptors; H_1_, excitatory H_1_ histamine receptors; 5HT_2A_ and 5HT_2C_, excitatory 5HT receptors. *Neuronal outputs*: excitatory (solid arrows) and inhibitory (broken arrows). The wakefulness-promoting nuclei exert a direct activating effect on the cerebral cortex; the VLPO promotes sleep by inhibiting the TMN and the LC. The LC promotes wakefulness by stimulating the cerebral cortex and the wakefulness-promoting neurones of the PPT, and by inhibiting the VLPO. The LC also inhibits the REM-sleep-promoting neurones of the PPT. The raphe nucleus promotes wakefulness by activating the cerebral cortex; this effect is attenuated by stimulation of GABAergic interneurones, which inhibit the LC and the VTA. The VTA exerts its wakefulness-promoting effect largely *via* activation of the LC, and the LH/PF largely *via* activation of the TMN and the LC. The connections of the LC are reviewed in detail in Part I. The GABAergic interneurones, activated by excitatory 5HT_2C_ receptors, are located in the VTA itself [55, 140] and in the vicinity of the LC [140]. Modified with permission from Szabadi, 2006.

**Fig. (2):**
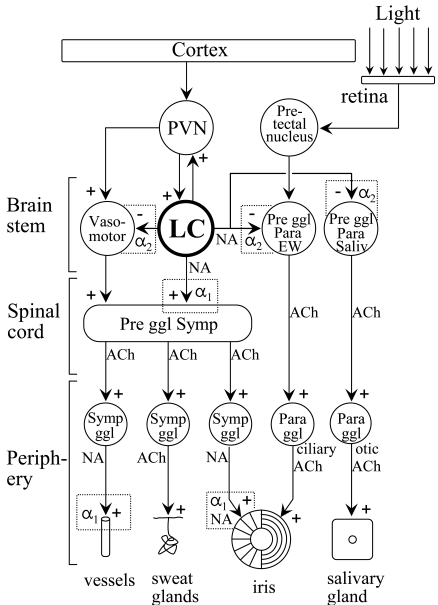
The central role of the locus coeruleus (LC) in the regulation of autonomic functions. *Nuclei*: PVN, paraventricular nucleus; Pre ggl Para, preganglionic parasympathetic neurones; EW, Edinger-Westphal nucleus; Saliv, salivatory nucleus; Pre ggl Symp, preganglionic sympathetic neurones; Symp ggl, sympathetic ganglion; Para ggl, parasympathetic ganglion. *Neurotransmitters*: NA, noradrenaline; ACh, acetylcholine. *Receptors*: α_1_ and α_2_, adrenoceptor subtypes. *Symbols*: +, excitatory, -, inhibitory. Organs comprising of smooth muscle (e.g., blood vessels, iris), or glandular tissue (e.g., sweat glands, salivary glands) receive autonomic (sympathetic and parasympathetic) innervations. Both innervations consist of a chain of two neurones (preganglionic and postganglionic) joined in a synapse located in the autonomic ganglion. Preganglionic sympathetic neurones are located in the intermediolateral cell column (IML) of the spinal cord whereas the preganglionic parasympathetic neurones are located in brainstem nuclei. Blood vessels (arterioles) and sweat glands receive sympathetic and salivary glands parasympathetic inputs whereas the smooth muscles in the iris are controlled by opposing sympathetic and parasympathetic inputs. The preganglionic neurones are always cholinergic, the postganglionic sympathetic neurones are noradrenergic, with the exception of those innervating the sweat glands which are cholinergic, whereas the postganglionic parasympathetic neurones are always cholinergic. The preganglionic neurones are influenced by premotor autonomic nuclei of which three are shown (PVN, vasomotor neurones located in the rostroventrolateral medulla, and the LC). The LC plays a pivotal role in autonomic regulation, influencing the activities of preganglionic neurones both directly and indirectly via the PVN and vasomotor neurones. The outputs from the LC can activate either excitatory α_1_-adrenoceptors or inhibitory α_2_-adrenoceptors. The output from the LC to the PVN is largely to its parvicellular subdivision; this connection plays a relatively minor role. The LC exerts an excitatory effect on preganglionic sympathetic neurones and an inhibitory effect on vasomotor premotor neurones and on preganglionic parasympathetic neurones. The activity of the preganglionic neurones is under the influence of the cerebral cortex. The light reflex is a parasympathetically-mediated reflex consisting of the constriction of the pupil in response to a light stimulus reaching the retina. The neuronal chain in the reflex includes the pretectal nucleus, the EW, and the ciliary ganglion. The connections of the LC shown in this figure are discussed in detail in Part I.

**Fig. (3):**
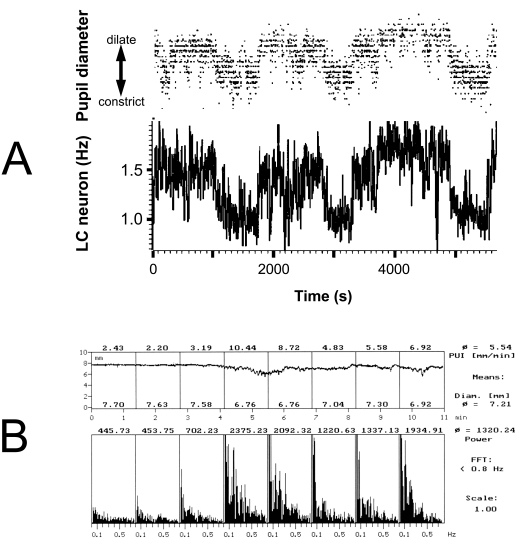
**A:** Relationship between pupil diameter and the firing rate of an LC neurone in a monkey. The two recordings were taken simultaneously. There was a clear parallelism between fluctuations in pupil diameter and firing rate. Reproduced with permission from Aston-Jones and Cohen (2005). **B:** An example recording from the pupillographic sleepiness test (PST) demonstrating fluctuations in resting pupil diameter (top) and the power spectrum (bottom) over an 11-minute recording period. The data for the total time period are divided into eight equal bins for further analysis. The fluctuations in *resting pupil diameter* are used in the analysis of the pupillary unrest index (PUI), the distance for which the margin of the pupil travels in one minute. The mean value of PUI obtained for the whole recording period is shown on the right of the figure. Vertical axis: pupil diameter (mm), horizontal axis: time (min). The mean pupil diameter for each bin is displayed above the horizontal axis, with the average diameter over the total recording period shown on the right of the figure. The mean PUI for each bin is displayed above the recording. The *power spectrum* is used in a Fast Fourier Transform analysis to derive a measure of total power (arbitrary units), shown on the right of the figure. Power (arbitrary units) is displayed along the vertical axis and frequency (Hz) is displayed along the horizontal axis for each time-bin individually. The total power for each bin is displayed above the power spectrum.

**Fig. (4):**
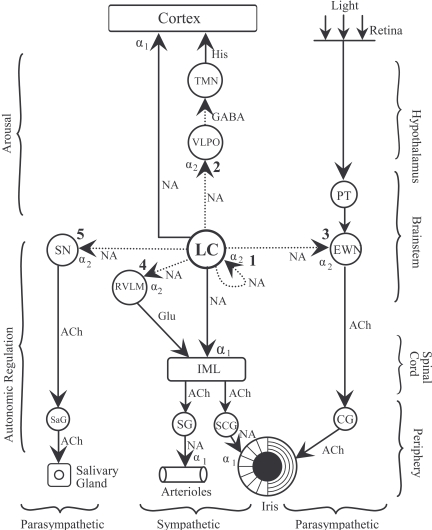
The central sites of action of α_2_-adrenoceptor agonists (e.g., clonidine, dexmedetomidine). *Nuclei*: TMN, tuberomamillary nucleus; VLPO, ventrolateral preoptic area; LC, locus coeruleus; PT, pretectal nucleus; SN, salivatory nucleus; EWN, Edinger-Westphal nucleus; RVLM, rostroventrolateral medulla; IML, intermediolateral cell column; SaG, salivary ganglion; SG, sympathetic ganglion; SCG, superior cervical ganglion; CG, ciliary ganglion. *Neurotransmitters*: NA, noradrenaline; GABA, γ-aminobutyric acid; Glu, glutamate; ACh, acetylcholine. *Receptors*: α_1_ and α_2_, adrenoceptor subtypes. Neuronal connections are indicated by arrows: solid lines, excitatory; dotted lines, inhibitory. The sites at which the LC exerts an inhibitory influence are indicated by numbers: 1. autoreceptors on LC neurones, 2. VLPO, 3. EWN, 4. RVLM, 5. SN. For the effects of the consequences of alterations of LC activity on arousal and autonomic function, see text.

**Fig. (5):**
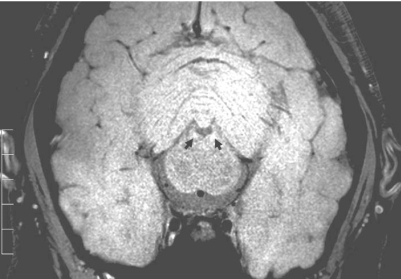
Magnetic resonance image showing a cross-section of the upper pons displaying the loci coerulei (LC). A picture was taken in a healthy human subject with the modification of the method of Sasaki *et al*., 2006, on a 3-tesla scanner to obtain neuromelanin signal to identify LC. The loci coerulei are shown by the small white areas in the bottom corners of the fourth cerebral ventricle, indicated by black arrows. By courtesy of Professor D. Auer, Queen’s Medical Centre, Nottingham.

**Fig. (6):**
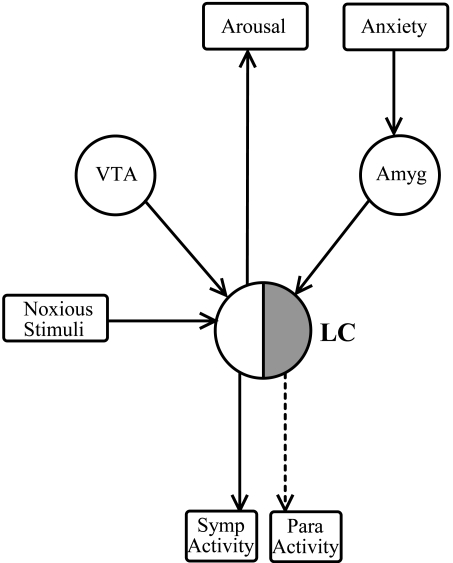
Schematic diagram to illustrate the hypothesis of two populations of locus coeruleus (LC) neurones. Sympathetic premotor neurones (shown as blank area) are preferentially activated by noxious stimuli from collaterals of ascending pain pathways and the dopaminergic neurones of the ventral tegmental area (VTA) (meso-coerulear pathway), whereas parasympathetic premotor neurones (shown as shaded area) are preferentially activated by anxiety *via* an output to the LC from the amygdala (Amyg). The sympathetic premotor neurones have a stimulatory effect on sympathetic activity (Symp Activity) and level of arousal whereas the parasympathetic premotor neurones exert an inhibitory influence on parasympathetic activity (Para Activity) (see text for details).

**Table 1:** Effects of α_2_-Adrenoceptor Agonists on Central Noradrenergic Pathways

Pathway	α_2_-Adrenoceptors Activated		Effect on Pathway	Effect Mediated by Pathway	Net Effect
	Pre-	Post-			
LC → VLPO	++	+	Inhibition ↓	Arousal ↓	Arousal ↓
LC → Cortex	++	0	Stimulation ↓	Arousal ↓	
LC → EWN^1^	++	+	Inhibition ↓	Pupil Diameter ↓	Pupil Diameter ↓
LC → IML^1^	++	0	Stimulation ↓	Pupil Diameter ↓	
LC → EWN^2^	+	++	Inhibition ↑	Pupil Diameter ↑	Pupil Diameter ↑
LC → IML^2^	++	0	Stimulation ↓	Pupil Diameter ↓	
LC → RVLM	++	+++	Inhibition ↑	Blood Pressure ↓	Blood Pressure ↓
LC → IML	++	0	Stimulation ↓	Blood Pressure ↓	
LC → Saliv. Nucl.	++	+++	Inhibition ↑	Salivation ↓	Salivation ↓

1 In man, dog and rabbit

2 In cat, rat and mouse

Abbreviations:

EWN    Edinger-Westphal nucleus IML     intermediolateral cell column of the spinal cord LC     locus coeruleus RVLM   rostroventrolateral medulla Saliv. Nucl. salivatory nuclei VLPO    ventrolateral preoptic nucleus Pre       pre-synaptic Post     post-synaptic

**Table 2:** Effects of Noradrenaline Reuptake Inhibitors on Central Noradrenergic Pathways

Pathway	Effect on Pathway	Effect Mediated by Pathway	Net Effect
LC → VLPO	Inhibition ↑	Arousal ↑	Arousal ↑
LC → Cortex	Stimulation ↑	Arousal ↑	
LC → EWN	Inhibition ↑	Pupil Diameter ↑	Pupil Diameter ↑
LC → IML	Stimulation ↑	Pupil Diameter ↑	
LC → RVLM	Inhibition ↑	Blood Pressure ↓	Blood Pressure ↑ (minor change)
LC → IML	Stimulation ↑	Blood Pressure ↑	
LC → Saliv. Nucl.	Inhibition ↑	Salivation ↓	Salivation ↓

Abbreviations:

EWN    Edinger-Westphal nucleus IML      intermediolateral cell column of the spinal cord LC        locus coeruleus RVLM    rostroventrolateral medulla Saliv. Nucl. salivatory nuclei VLPO    ventrolateral preoptic nucleus

**Table 3:** Effects of α2-Adrenoceptor and µ-Opiate Receptor Agonists on Arousal and Pupil Diameter

		Arousal	Pupil Diameter
**Pre-synaptic Action Dominant**			
Man	α_2_-adrenoceptor agonist	↓ [182]	↓ [39, 76, 184, 341, 379]
	µ-opiate receptor agonist	↓ [131]	↓ [225, 286, 334, 343, 344, 345]
Dog	α_2_-adrenoceptor agonist	↓ [84]	↓ [396]
	µ-opiate receptor agonist	↓ [502]	↓ [396]
Rabbit	α_2_-adrenoceptor agonist	no information available	↓ [8]
	µ-opiate receptor agonist	↓ [278]	↓ [450]
**Post-synaptic Action Dominant**			
Cat	α_2_-adrenoceptor agonist	↑ [245]	↑ [231, 232]
	µ-opiate receptor agonist	↑ [95, 221]	↑ [80]
Rat	α_2_-adrenoceptor agonist	↑ [357]	↑ [137, 166, 169, 175, 231]
	µ-opiate receptor agonist	↑ [12, 484]	↑ [224]
Mouse	α_2_-adrenoceptor agonist	↑ [159]	↑ [167, 168]
	µ-opiate receptor agonist	↑ [215]	↑ [230]

References are indicated in square brackets.

## Abbreviations

6-OHDA, 6-hydroxydopamine; AD, Alzheimer’s disease; BF, Basal forebrain; CR, Caudal raphe; CS, Conditioned stimulus; DMV, Dorsal motor nucleus of the vagus; DR, Dorsal raphe; EDS, Excessive daytime sleepiness; EEG, Electroencephalogram; EMG, Electromyogram; EWN, Edinger-Westphal nucleus; fMRI, Functional magnetic resonance imaging; GABA, Gamma-aminobutyric acid; LC, Locus coeruleus; LDT, Laterodorsal tegmental nucleus; MPTP, 1-methyl-4-phenyl- 1,2,3,6- tetrahydropyridine; PD, Parkinson’s disease; PPT, Pedunculopontine tegmental nucleus; PST, Pupillographic sleepiness test; PVN, Paraventricular nucleus; REM, Rapid eye movement; RVLM, Rostroventrolateral medulla; SWS, Slow wave sleep; US, Unconditioned stimulus; VLPO, Ventrolateral preoptic area; VTA, Ventral tegmental area.

## References

[1] Aantaa R (1991). Assessment of the sedative effects of dexmedetomidine, an alpha 2-adrenoceptor agonist, with analysis of saccadic eye movements. Pharmacol. Toxicol.

[2] Abdelmawla AH, Langley RW, Szabadi E, Bradshaw CM (1999). Comparison of the effects of venlafaxine, desipramine, and paroxetine on noradrenaline- and methoxamine-evoked constriction of the dorsal hand vein. Br. J. Clin. Pharmacol.

[3] Abduljawad KAJ, Langley RW, Bradshaw CM, Szabadi E (1997). Effects of clonidine and diazepam on the acoustic startle response and on its inhibition by ‘prepulses’ in man. J. Psychopharmacol.

[4] Abduljawad KAJ, Langley RW, Bradshaw CM, Szabadi E (2001). Effects of clonidine and diazepam on prepulse inhibition of the acoustic startle response and the N1/P2 auditory evoked potential in man. J. Psychopharmacol.

[5] Abercrombie ED, Jacobs BL (1987). Microinjected clonidine inhibits noradrenergic neurons of the locus coeruleus in freely moving cats. Neurosci. Lett.

[6] Aghajanian GK, VanderMaelen CP (1982). Alpha 2-adreno-ceptor- mediated hyperpolarization of locus coeruleus neurons: intracellular studies *in vivo*. Science.

[7] Akaoka H, Roussel B, Lin JS, Chouvet G, Jouvet M (1991). Effect of modafinil and amphetamine on the rat catecholaminergic neuron activity. Neurosci. Lett.

[8] Allen RC, Langman ME (1976). The intra-ocular pressure response of conscious rabbits to clonidine. Invest. Ophthalmol.

[9] Almeida MC, Steiner AA, Coimbra NC, Branco LGS (2004). Thermoeffector neuronal pathways in fever: a study in rats showing a new role for the locus coeruleus. J. Physiol.

[10] Amara SG, Kuhar MJ (1993). Neurotransmitter transporters: Recent progress. Ann. Rev. Neurosci.

[11] Ameli R, Ip C, Grillon C (2001). Contextual fear-potentiated startle conditioning in humans: Replication and extension. Psychophysiology.

[12] Arankowsky-Sandoval G, Gold PE (1995). Morphine-induced deficits in sleep patterns: Attenuation by glucose. Neurobiol. Learn. Mem.

[13] Arima K, Akashi T (1990). Involvement of the locus coeruleus in Pick’s disease with or without Pick body formation. Acta Neuropathologica.

[14] Arnulf I, Konofal E, Merino-Andreu M, Houeto JL, Mesnage V, Welter ML, Lacomblez L, Golmard JL, Derenne JP, Agid Y (2002). Parkinson’s disease and Sleepiness. Neurology.

[15] Arya DK, Langley RW, Szabadi E, Bradshaw CM (1997). Comparison of the effects of high ambient temperature and clonidine on autonomic functions in man. Naunyn Schmiedeberg’s Arch. Pharmacol.

[16] Aston-Jones G (2005). Brain structures and receptors involved in alertness. Sleep. Med.

[17] Aston-Jones G, Bloom FE (1981). Activity of norepinephrine-containing locus coeruleus neurons in behaving rats anticipates fluctuations in the sleep-waking cycle. J. Neurosci.

[18] Aston-Jones G, Chiang C, Alexinsky T (1991). Discharge of noradrenergic locus coeruleus neurons in behaving rats and monkeys suggests a role in vigilance. Prog. Brain Res.

[19] Aston-Jones G, Cohen JD (2005). An integrative theory of locus coeruleus-norepinephrine function: adaptive gain and optimal performance. Ann. Rev. Neurosci.

[20] Aston-Jones G, Hirata H, Akaoka H (1997). Local opiate withdrawal in locus coeruleus *in vivo*. Brain Res.

[21] Bagetta G, De Sarro G, Priolo E, Nistico G (1988). Ventral tegmental area: site through which dopamine D_2_-recepor agonists evoke behavioural and electrocortical sleep in rats. Br. J. Pharmacol.

[22] Bakes A, Bradshaw CM, Szabadi E (1990). Attenuation of the pupillary light reflex in anxious patients. Br. J. Clin. Pharmacol.

[23] Baloyannis SJ, Costa V, Baloyannis IS (2006). Morphological alterations of the synapses in the locus coeruleus in Parkinson’s disease. J. Neurol. Sci.

[24] Barrett SL, Bell R, Watson D, King DJ (2004). Effects of amisulpride, risperidone and chlorpromazine on auditory and visual latent inhibition, prepulse inhibition, executive function and eye movements in healthy volunteers. J. Psychopharmacol.

[25] Barry RJ, Rushby JA, Wallace MJ, Clarke AR, Johnstone SJ, Zlojutro I (2005). Caffeine effects on resting-state arousal. Clin. Neurophysiol.

[26] Bay KD, Mamiya K, Good CH, Skinner RD, Garcia-Rill E (2006). Alpha-2 adrenergic regulation of pedunculopontine nucleus neurons during development. Neuroscience.

[27] Benarroch EE, Bannister R, Mathias CJ (1992). Central neurotransmitters and neuromodulators in cardiovascular regulation Autonomic Failure. Autonomic Failure.

[28] Bennett HJ, Semba K (1998). Immunohistochemical localization of caffeine-induced c-Fos protein expression in the rat brain. J. Comp. Neurol.

[29] Berridge CW, Foote SL (1991). Effects of locus coeruleus activation on electroencephalographic activity in neocortex and hippocampus. J. Neurosci.

[30] Berridge CW, Page ME, Valentino RJ, Foote SL (1993). Effects of locus coeruleus activation on electroencephalographic activity in neocortex and hippocampus. Neuroscience.

[31] Berrocoso E, Micó JA, Ugedo L (2006). *In vivo* effect of tramadol on locus coeruleus neurons is mediated by α_2_-adrenoceptors and modulated by serotonin. Neuropharmacology.

[32] Bertrand E, Lechowicz W, Szpak GM, Dymecki J (1997). Qualitative and quantitative analysis of locus coeruleus neurons in Parkinson’s disease. Folia Neuropathol.

[33] Bezard E, Brefel C, Tison F, Peyro-Saint-Paul H, Ladure P, Rascol O, Gross CE (1999). Effect of the α2 adrenoreceptor antagonist, idazoxan, on motor disabilities in MPTP-treated monkey. Prog. Neuro-Psychopharmacol. Biol. Psychiatry.

[34] Bickford-Wimer PC, Parfitt K, Hoffer BJ, Freedman R (1987). Desipramine and noradrenergic neurotransmission in aging: failure to respond in aged laboratory animals. Neuropharmacology.

[35] Bing G, Zhang Y, Watanabe Y, McEwen BS, Stone EA (1994). Locus coeruleus lesions potentiate neurotoxic effects of MPTP in dopaminergic neurons of the substantia nigra. Brain Res.

[36] Bitsios P, Philpott A, Langley RW, Bradshaw CM, Szabadi E (1999). Comparison of the effects of diazepam on the fear-potentiated startle reflex and the fear-inhibited light reflex in man. J. Psychopharmacol.

[37] Bitsios P, Prettyman R, Szabadi E (1996). Changes in autonomic function with age: A study of pupillary kinetics in healthy young and old people. Age Ageing.

[38] Bitsios P, Szabadi E, Bradshaw CM (1996). The inhibition of the light reflex by the threat of an electric shock: a potential laboratory model of human anxiety. J. Psychopharmacol.

[39] Bitsios P, Szabadi E, Bradshaw CM (1998). The effects of clonidine on the fear-inhibited light reflex. J. Psychopharmacol.

[40] Bitsios P, Szabadi E, Bradshaw CM (1998). Sensitivity of the fear- inhibited light reflex to diazepam. Psychopharmacology.

[41] Bitsios P, Szabadi E, Bradshaw CM (1999). Comparison of the effects of venlafaxine, paroxetine and desipramine on the pupillary light reflex in man. Psychopharmacology.

[42] Bitsios P, Szabadi E, Bradshaw CM (2004). The fear-inhibited light reflex: importance of the anticipation of an aversive event. Int. J. Psychophysiol.

[43] Bogdanski DF, Sulser F, Brodie BB (1961). Comparative action of reserpine, tetrabenazine and chlorpromazine on central parasympathetic activity: effects on pupillary size and lacrimation in rabbit and on salivation in dog. J. Pharmacol. Exp. Ther.

[44] Bondareff W, Mountjoy CQ, Roth M, Rossor MN, Iverson LL, Reynolds GP, Hauser DL (1987). Neuronal degeneration in locus coeruleus and cortical correlates of Alzheimer disease. Alzheimer Dis. Assoc. Disord.

[45] Bostock MI (1975). A clinical trial of clonidine (Catapres) in private practice. N. Z. Med. J.

[46] Bousquet P, Greney H, Bruban V, Schann S, Ehrhardt JD, Monassier L, Feldman J (2003). I(1) imidazoline receptors involved in cardiovascular regulation: where are we and where are we going?. Ann. N.Y. Acad. Sci.

[47] Bousquet P, Guertzenstein PG (1973). Localization of the central cardiovascular action of clonidine. Br. J. Pharmacol.

[48] Boutrel B, Koob GF (2004). What keeps us awake: the neuropharmacology of stimulants and wakefulness-promoting medications. Sleep.

[49] Braak H, Del Tredici K, Rüb U, de Vos RAI, Jansen Steur ENH, Braak E (2003). Staging of brain pathology related to sporadic Parkinson’s disease. Neurobiol. Aging.

[50] Breen LA, Burde RM, Loewy AD (1983). Brainstem connections to the Edinger-Westphal nucleus of the cat: a retrograde tracer study. Brain Res.

[51] Bremner JD, Krystal JH, Southwick SM, Charney DS (1996). Noradrenergic mechanisms in stress and anxiety: I. preclinical studies. Synapse.

[52] Brodal P (2004). The Central Nervous System.

[53] Brownstein MJ, Hoffman BJ (1994). Neurotransmitter transporters. Recent Prog. Horm. Res.

[54] Bruce M, Scott N, Lader M, Marks V (1986). The psychopharmacological and electrophysiological effects of single doses of caffeine in healthy human subjects. Br. J. Clin. Pharmacol.

[55] Bubar MJ, Cunningham KA (2007). Distribution of serotonin 5-HT_2C_ receptors in the ventral tegmental area. Neuroscience.

[56] Bullitt E (1990). Expression of c-fos-like protein as a marker for neuronal activity following noxious stimulation in the rat. J. Comp. Neurol.

[57] Burke WJ, Li SW, Schmitt CA, Xia P, Chung HD, Gillespie KN (1999). Accumulation of 3,4-dihydroxyphenylglycolal-dehyde, the neurotoxic monoamine oxidase A metabolite of norepinephrine, in locus ceruleus cell bodies in Alzheimer’s disease: mechanism of neuron death. Brain Res.

[58] Busch C, Bohl J, Ohm TG (1997). Spatial, temporal and numeric analysis of Alzheimer changes in the nucleus coeruleus. Neurobiol. Aging.

[59] Caldwell JA Jr (1996). Effects of operationally effective doses of dextroamphetamine on heart rates and blood pressures of army aviators. Mil. Med.

[60] Cash R, Dennis T, L'Heureux R, Raisman R, Javoy-Agid F, Scatton B (1987). Parkinson’s disease and dementia: norepinephrine and dopamine in locus coeruleus. Neurology.

[61] Cedarbaum JM, Aghajanian GK (1978). Afferent projections to the rat locus coeruleus as determined by a retrograde tracing technique. J. Comp. Neurol.

[62] Cespuglio R, Rousset C, Debilly G, Rochat C, Millan MJ (2005). Acute administration of the novel serotonin and noradrenaline reuptake inhibitor, S33005, markedly modifies sleep-wake cycle architecture in the rat. Psychopharmacology (Berl.).

[63] Chan-Palay V (1991). Alterations in the locus coeruleus in dementias of Alzheimer’s and Parkinson’s disease. Prog. Brain Res.

[64] Chan-Palay V, Asan E (1989). Alterations in catecholamine neurons of the locus coeruleus in senile dementia of the Alzheimer’s type and in Parkinson’s disease with and without dementia and depression. J. Comp. Neurol.

[65] Chan-Palay V, Asan E (1989). Quantitation of catecholamine neurons in the locus coeruleus in human brains of normal young and older adults and in depression. J. Comp. Neurol.

[66] Chapman CR, Oka S, Bradshaw DH, Jacobson RC, Donaldson GW (1999). Phasic pupil dilation response to noxious stimulation in normal volunteers: Relationship to brain evoked potentials and pain report. Psychophysiology.

[67] Charney DS, Deutch A (1996). A functional neuroanatomy of anxiety and fear: implications for the pathophysiology and treatment of anxiety disorders. Crit. Rev. Neurobiol.

[68] Charney DS, Redmond DE Jr (1983). Neurobiological mechanisms in human anxiety. Neuropharmacology.

[69] Charney DS, Woods SW, Nagy LM, Southwick SM, Krystal JH, Heninger GR (1990). Noradrenergic function in panic disorder. J. Clin. Psychiatry.

[70] Chen F-J, Sara SJ (2007). Locus coeruleus activation by foot shock or electrical stimulation inhibits amygdala neurons. Neuroscience.

[71] Chen CL, Yang YR, Chiu TH (1999). Activation of rat locus coeruleus neuron GABA(A) receptors by propofol and its potentiation by pentobarbital or alphaxalone. Eur. J. Pharmacol.

[72] Cheng S-Y, Glazkova D, Serova L, Sabban EL (2005). Effect of prolonged nicotine infusion on response of rat catecholamine biosynthetic enzymes to restraint and cold stress. Pharmacol. Biochem. Behav.

[73] Chiu TH, Chen MJ, Yang YR, Yang JJ, Tang FI (1995). Actions of dexmedetomidine on rat locus coeruleus neurones: intracellular recording *in vitro*. Eur. J. Pharmacol.

[74] Chou TC, Bjorkum AA, Gaus SE, Lu J, Scammell TE, Saper CB (2002). Afferents to the ventrolateral preoptic nucleus. J. Neurosci.

[75] Christie MJ (1991). Mechanisms of opioid actions on neurons of the locus coeruleus. Prog. Brain Res.

[76] Clifford JM, Day MD, Orwin JM (1982). Reversal of clonidine induced miosis by the alpha 2-adrenoceptor antagonist RX 781094. Br. J. Clin. Pharmacol.

[77] Coffman JD (1992). α2-adrenergic and serotonergic receptors and forearm venous compliance in normal human subjects. J. Cardiovasc. Pharmacol.

[78] Collier TJ, Greene JG, Felten DL, Stevens SY, Steece Collier K (2004). Reduced cortical noradrenergic neurotransmission is associated with increased neophobia and impaired spatial memory in aged rats. Neurobiol. Aging.

[79] Collingridge GL, James TA, MacLeod NK (1979). Neurochemical and electrophysiological evidence for a projection from the locus coeruleus to the substantia nigra. J. Physiol.

[80] Corpas I, de Andrés I (1991). Morphine effects in brainstem-transected cats: I EEG and ‘sleep-wakefulness’ in the isolated forebrain. Behav. Brain Res.

[81] Coupland NJ, Bailey JE, Wilson SJ, Potter WZ, Nutt DJ (1994). A pharmacodynamic study of the alpha 2-adrenergic receptor antagonist ethoxidazoxan in healthy volunteers. Clin. Pharmacol. Ther.

[82] Craig AD (1992). Spinal and trigeminal lamina I input to the locus coeruleus anterogradely labeled with Phaseolus vulgaris leucoagglutinin (PHA-L) in the cat and monkey. Brain Res.

[83] Cui ZT, Zhang TM, Su ZH, Yen WW (1988). Morphological changes in locus coeruleus of albino rats in relation to aging. Acta Anat. (Basel).

[84] Cullen LK (1996). Medetomidine sedation in dogs and cats: a review of its pharmacology, antagonism and dose. Br. Vet. J.

[85] Cullinan WE, Herman JP, Battaglia DF, Akil H, Watson SJ (1995). Pattern and time course of immediate early gene expression in rat brain following acute stress. Neuroscience.

[86] Curet O, De Montigny C, Blier P (1992). Effect of desipramine and amphetamine on noradrenergic neurotransmission: electrophysiological studies in the rat brain. Eur. J. Pharmacol.

[87] Curran S, Wattis J (2000). Critical flicker fusion threshold: a potentially useful measure for the early detection of Alzheimer’s disease. Hum. Psychopharmacol. Clin. Exp.

[88] Curran S, Wilson S, Musa S, Wattis J (2004). Critical flicker fusion threshold in patients with Alzheimer’s disease and vascular dementia. Int. J. Geriatr. Psychiatry.

[89] Curtis AL, Conti E, Valentino RJ (1993). Cocaine effects on brain noradrenergic neurons of anesthetized and unanesthetized rats. Neuropharmacology.

[90] Damasio AR (1998). Emotion in the perspective of an integrated nervous system. Brain Res. Rev.

[91] Danysz W, Dyr W, Plaznik A, Kostowski W (1989). The effect of microinjections of clonidine into the locus coeruleus on cortical EEG in rats. Pol. J. Pharmacol. Pharm.

[92] Davies MF, Tsui JY, Flannery JA, Li X, DeLorey TM, Hoffman BB (2003). Augmentation of the noradrenergic system in alpha-2 adrenergic receptor deficient mice: anatomical changes associated with enhanced fear memory. Brain Res.

[93] Davis M (1998). Are different parts of the extended amygdala involved in fear versus anxiety?. Biol. Psychiatry.

[94] Davis M, Redmond DE, Baraban JM (1979). Noradrenergic agonists and antagonists: effects on conditioned fear as measured by the potentiated startle paradigm. Psychopharmacology (Berl.).

[95] de Andrés I, Corpas I (1991). Morphine effects in brainstem-transected cats II. Behavior and sleep of the decerebrate cat. Behav. Brain Res.

[96] DeBattista C, Doghramji K, Menza MA, Rosenthal MH, Fieve RR Modafinil in Depression Study Group (2003) Adjunct modafinil for the short-term treatment of fatigue and sleepiness in patients with major depressive disorder: a preliminary double-blind, placebo-controlled study. J. Clin. Psychiatry.

[97] Del Tredici K, Rub U, De Vos RA, Bohl JR, Braak H (2002). Where does Parkinson disease pathology begin in the brain?. J Neuropathol. Exp. Neurol.

[98] De Maio D, Johnson FN (2000). The clinical efficacy of reboxetine in the treatment of depression. Rev. Contemp. Pharmacother.

[99] De Sarro GB, Bagetta G, Ascioti C, Libri V, Nistico G (1988). Microinfusion of clonidine and yohimbine into locus coeruleus alters EEG power spectrum: effects of aging and reversal by phosphatidylserine. Br. J. Pharmacol.

[100] Deurveilher S, Lo H, Murphy JA, Burns J, Semba K (2006). Differential c-Fos immunoreactivity in arousal-promoting cell groups following systemic administration of caffeine in rats. J. Comp. Neurol.

[101] Dishman RK, Renner KJ, White-Welkley JE, Burke KA, Burmell BN (2000). Treadmill exercise training augment brain norepinephrine response to familiar and novel stress. Brain Res. Bull.

[102] Dopheide MM, Morgan RE, Rodvelt KR, Schachtman TR, Miller DK (2007). Modafinil evokes striatal [^3^H]dopamine release and alters the subjective properties of stimulants. Eur. J. Pharmacol.

[103] Dorr AE, Debonnel G (2006). Effect of vagus nerve stimulation of serotonergic and noradrenergic transmission. J. Pharmacol. Exp. Ther.

[104] Dortch-Carnes J, Russell KR (2006). Morphine-induced reduction of intraocular pressure and pupil diameter: role of nitric oxide. Pharmacology.

[105] Downs JL, Dunn MR, Borok E, Shanabrough M, Horvath TL, Kohama SG, Urbanski HF (2007). Orexin neuronal changes in the locus coeruleus of the aging rhesus macaque. Neurobiol. Aging.

[106] Drolet G, Gauthier P (1985). Peripheral and central mechanisms of the pressor response elicited by stimulation of the locus coeruleus in the rat. Can. J. Physiol. Pharmacol.

[107] Eiden LE (2000). The vesicular neurotransmitter transporters: current perspectives and future prospects. FASEB J.

[108] Ekimova IV (2003). Changes in the metabolic activity of neurons in the anterior hypothalamic nuclei in rats during hyperthermia, fever, and hypothermia. Neurosci. Behav. Physiol.

[109] Elam M, Svensson TH, Thoren P (1986). Locus coeruleus neurons and sympathetic nerves: activation by cutaneous sensory afferents. Brain Res.

[110] Elrod R, Peskind ER, DiGiacomo L, Brodkin KI, Veith RC, Raskind MA (1997). Effects of Alzheimer’s disease severity on cerebrospinal fluid norepinephrine concentration. Am. J. Psychiatry.

[111] España RA, Berridge CW (2006). Organisation of noradrenergic efferents to arousal-related basal forebrain structures. J. Comp. Neurol.

[112] Fabris G, Anselmo-Franci JA, Branco LG (1999). Role of nitric oxide in hypoxia-induced hyperventilation and hypothermia: participation of the locus coeruleus. Braz. J. Med. Biol. Res.

[113] Featherby T, Lawrence AJ (2004). Chronic cold stress regulates ascending noradrenergic pathways. Neuroscience.

[114] Fernández-Pastor B, Mateo Y, Gómez-Urquijo S, Meana JJ (2005). Characterization of noradrenaline release in the locus coeruleus of freely moving awake rats by *in vivo* microdialysis. Psychopharmacology (Berl).

[115] Fernández-Pastor B, Meana JJ (2002). *In vivo* tonic modulation of the noradrenaline release in the rat cortex by locus coeruleus somatodendritic α_2_-adrenoceptors. Eur. J. Pharmacol.

[116] Ferreira JJ, Galitzky M, Thalamus C, Tiberge M, Montastruc JL, Sampaio C, Rascol O (2002). Effect of ropinirole on sleep onset: a randomised, placebo-controlled study in healthy volunteers. Neurology.

[117] Fisone G, Borgkvist A, Usiello A (2004). Caffeine as a psychomotor stimulant: mechanism of action. Cell Mol. Life Sci.

[118] Fletcher WA, Sharpe JA (1986). Saccadic eye movement dysfunction in Alzheimer’s disease. Ann. Neurol.

[119] Fletcher WA, Sharpe JA (1988). Smooth pursuit dysfunction in Alzheimer’s disease. Neurology.

[120] Fodor M, Görcs TJ, Palkovits M (1992). Immunohistochemical study on the distribution of neuopeptides within the pontine tegmentum - particularly the parabrachial nuclei and the locus coeruleus of the human brain. Neuroscience.

[121] Foote SL, Aston-Jones GS, Bloom FE, Kupfer DJ (1995). Pharmacology and Physiology of Central Noradrenergic Systems. Psychopharmacology The Fourth Generation of Progress.

[122] Foote SL, Aston-Jones G, Bloom FE (1980). Impulse activity of locus coeruleus neurons in awake rats and monkeys is a function of sensory stimulation and arousal. Proc. Natl. Acad. Sci. USA.

[123] Foote SL, Berridge CW, Adams LM, Pineda JA (1991). Electrophysiological evidence for the involvement of the locus coeruleus in alerting, orienting, and attending. Prog. Brain Res.

[124] Foote SL, Bloom FE, Aston-Jones G (1983). Nucleus locus coeruleus: new evidence of anatomical and physiological specificity. Physiol. Rev.

[125] Forstl H, Levy R, Burns A, Luthert P, Cairns N (1994). Disproportionate loss of noradrenergic and cholinergic neurons as cause of depression in Alzheimer’s disease- a hypothesis. Pharmacopsychiatry.

[126] Fort P, Khateb A, Pegna A, M?hlethaler M, Jones BE (1995). Noradrenergic modulation of cholinergic nucleus basalis neurons demonstrated by *in vitro* pharmacological and immunohistochemical evidence in the guinea-pig brain. Eur. J. Neurosci.

[127] Fyhrquist F, Kurppa K, Huuskonen M (1975). Plasma renin activity, blood pressure and sodium excretion during treatment with clonidine. Acta Med. Scan.

[128] Gallopin T, Luppi PH, Rambert FA, Frydman A, Fort P (2004). Effect of the wake-promoting agent modafinil on sleep-promoting neurons from the ventrolateral preoptic nucleus: an *in vitro* pharmacologic study. Sleep.

[129] Gallopin T, Luppi PH, Cauli B, Urade Y, Rossier J, Hayaishi O, Lanbolez B, Fort P (2005). The endogenous somnogen adenosine excites a subset of sleep-promoting neurons *via* A2A receptors in the ventrolateral preoptic nucleus. Neuroscience.

[130] Garcia C, Schmitt P, D'Aleo P, Bittel J, Cure M, Pujol JF (1994). Regional specificity of the long-term variation of tyrosine hydroxylase protein in rat catecholaminergic cell groups after chronic heat exposure. J. Neurochem.

[131] Garzon M, Tejero S, Beneitez AM, de Andres I (1995). Opiate microinjections in the locus coeruleus area of the cat enhance slow wave sleep. Neuropeptides.

[132] German DC, Liang C-L, Manaye KF, Lane K, Sonsalia PK (2000). Pharmacological inactivation of the vesicular monoamine transporter can enhance 1-methyl-4-phenyl-1, 2, 3, 6-tetrahydropyridine-induced neurodegeneration of midbrain dopaminergic neurons, but not locus coeruleus noradrenergic neurons. Neuroscience.

[133] German DC, Manaye KF, White CL 3rd, Woodward DJ, McIntire DD, Smith WK, Kalaria RN, Mann DM (1992). Disease-specific patterns of locus coeruleus cell loss. Ann. Neurol.

[134] German DC, Nelson O, Liang F, Liang C-L, Games D (2005). The PDAPP mouse model of Alzheimer's disease: locus coeruleus neuronal shrinkage. J. Comp. Neurol.

[135] Gertler R, Brown HC, Mitchell DH, Silvius EN (2001). Dexmedetomidine: a novel sedative-analgesic agent. Proc. (Bayl. Univ. Med. Cent.).

[136] Gesi M, Soldani P, Giorgi FS, Santinami A, Bonaccorsi I, Fornai F (2000). The role of locus coeruleus in the development of Parkinson’s disease. Neurosci. Biobehav. Rev.

[137] Gherezghiher T, Koss MC (1979). Clonidine mydriasis in the rat. Eur. J. Pharmacol.

[138] Glue P, Nutt D (1988). Clonidine challenge testing of alpha-2- adrenoceptor function in man: the effects of mental illness and psychotropic medication. J. Psychopharmacol.

[139] Glue P, White E, Wilson S, Ball DM, Nutt DJ (1991). Pharmacology of saccadic eye movements in man. 2. Effects of the alpha 2-adrenoceptor ligands idazoxan and clonidine. Psychopharmacology (Berl.).

[140] Gobert A, Rivet J-M, Lejeune F, Newman-Tancredi A, Adhumeau- Auclair A, Nicolas J-P, Cistarelli L, Melon C, Millan MJ (2000). Serotonin_2C_ receptors tonically suppress the activity of mesocortical dopaminergic and adrenergic, but not serotonergic, pathways: A combined dialysis and electrophysiological analysis in the rat. Synapse.

[141] Goddard AW, Charney DS, Germine M, Woods SW, Heninger GR, Krystal JH, Goodman WK, Price LH (1995). Effects of tryptophan depletion on responses to yohimbine in healthy human subjects. Biol. Psychiatry.

[142] González MM, Debilly G, Valatx J-L (1998). Noradrenaline neurotoxin DSP-4 effects on sleep and brain temperature in the rat. Neurosci. Lett.

[143] Gowing LR, Farrell M, Ali RL, White JM (2002). Alpha2-adrenergic agonists in opioid withdrawal. Addiction.

[144] Graham SJ, Scaife JC, Langley RW, Bradshaw CM, Szabadi E, Xi L, Crumley T, Calder N, Gottesdiener K, Wagner JA (2005). Effects of lorazepam on fear-potentiated startle responses in man. J. Psychopharmacol.

[145] Grandoso L, Pineda J, Ugedo L (2004). Comparative study of the effects of desipramine and reboxetine on locus coeruleus neurons in rat brain slices. Neuropharmacology.

[146] Grenhoff J, Nisell M, Ferre S, Aston-Jones G, Svensson TH (1993). Noradrenergic modulation of midbrain dopamine cell firing elicited by stimulation of the locus coeruleus in the rat. J. Neural Transm. Gen. Sect.

[147] Grillon C, Cordova J, Levine LR, Morgan CA (2003). Anxiolytic effects of a novel group II metabotropic glutamate receptor agonist (LY354740) in the fear-potentiated startle paradigm in humans. Psychopharmacology (Berl.).

[148] Grossman E, Rosenthal T, Peleg E, Holmes C, Goldstein DS (1993). Oral yohimbine increases blood pressure and sympathetic nervous outflow in hypertensive patients. J. Cardiovasc. Pharmacol.

[149] Groves DA, Brown VJ (2005). Vagal nerve stimulation: a review of its applications and potential mechanisms that mediate its clinical effects. Neurosci. Biobehav. Rev.

[150] Grudzien A, Shaw P, Weintraub S, Bigio E, Mash DC, Mesulam MM (2007). Locus coeruleus neurofibrillary degeneration in aging, mild cognitive impairment and early Alzheimer’s disease. Neurobiol. Aging.

[151] Grunberger J, Saletu B, Linzmayer L, Barbanoj MJ (1993). Clinical- pharmacological study with the two isomers (d-, l-) of fenfluramine and its comparison with chlorpromazine and d-amphetamine: psychometric and psychophysiological evaluation. Methods Find. Exp. Clin. Pharmacol.

[152] Gurtu S, Pant KK, Sinha JN, Bhargava KP (1984). An investigation into the mechanism of cardiovascular responses elicited by electrical stimulation of locus coeruleus and subcoeruleus in the cat. Brain Res.

[153] Hajos M, Engberg G (1990). A role of excitatory amino acids in the activation of locus coeruleus neurons following cutaneous thermal stimuli. Brain Res.

[154] Haeusler G (1975). Cardiovascular regulation by central adrenergic mechanisms and its alteration by hypotensive drugs. Circ. Res.

[155] Hamburg M, Tallman J (1981). Chronic morphine administration increases the apparent number of alpha_2_-adrenergic receptors in rat brain. Nature.

[156] Hamilton C, van Zwieten PA, Julius S, Hamilton C.A, Prichard B.N.C (1996). Chemistry, Mechanism of Action and Experimental Pharmacology of Moxonidine. The I1-imidazoline Receptor Agonist Moxonidine: A New Antihypertensive.

[157] Hamilton MJ, Smith PR, Peck AW (1983). Effects of bupropion, nomifensine and dexamphetamine on performance, subjective feelings, autonomic variables and electroencephalogram in healthy volunteers. Br. J. Clin. Pharmacol.

[158] Han SK, Chong W, Li LH, Lee IS, Murase K, Ryu PD (2002). Noradrenaline excites and inhibits GABAergic transmission in parvocellular neurons of rat hypothalamic paraventricular nucleus. J. Neurophysiol.

[159] Handley SL, Thomas KV (1978). On the mechanism of amphetamine- induced behavioural changes in the mouse. II Effects of agents stimulating noradrenergic receptors. Arzneimittelforschung.

[160] Harron DW, Hasson B, Regan M, McClelland RJ, King DJ (1995). Effects of rilmenidine and clonidine on the electroencephalogram, saccadic eye movements, and psychomotor function. J. Cardiovasc. Pharmacol.

[161] Hauser RA, Gauger L, McDowell Anderson W, Zesiewicz TA (2000). Pramipexole-induced somnolence and episodes of daytime sleep. Mov. Disord.

[162] Haywood JR, Mifflin SW, Craig T, Calderon A, Hensler JG, Hinojosa-Laborde C (2001). gamma-Aminobutyric acid (GABA) - A function and binding in the paraventricular nucleus of the hypothalamus in chronic renal-wrap hypertension. Hypertension.

[163] Head GA, Chan CKS, Burke SL (1998). Relationship between imidazoline and α 2-adrenoceptors involved in the sympatho-inhibitory actions of centrally acting antihypertensive agents. J. Auton. Nerv. Syst.

[164] Head GA, Gundlach AL, Musgrave IF (1998). Recent advances in imidazoline receptor research: ligands - localization and isolation â€“ signalling - functional and clinical studies. J. Auton. Nerv. Syst.

[165] Head GA, Mayorov DN (2006). Imidazoline receptors, novel agents and therapeutic potential. Cardiovasc. Hematol. Agents Med. Chem.

[166] Heal DJ, Cheetham SC, Butler SA, Gosden J, Prow MR, Buckett WR (1995). Receptor binding and functional evidence suggest that post-synaptic alpha 2-adrenoceptors in rat brain are of the alpha 2D subtype. Eur. J. Pharmacol.

[167] Heal DJ, Prow MR, Buckett WR (1989). Clonidine produces mydriasis in conscious mice by activating central alpha 2-adrenoceptors. Eur. J. Pharmacol.

[168] Heal DJ, Prow MR, Buckett WR (1989). Clonidine-induced hypoactivity and mydriasis in mice are respectively mediated *via* pre- and postsynaptic alpha 2-adrenoceptors in the brain. Eur. J. Pharmacol.

[169] Heal DJ, Prow MR, Butler SA, Buckett WR (1995). Mediation of mydriasis in conscious rats by central postsynaptic α_2_-adrenoceptors. Pharmacol. Biochem. Behav.

[170] Heider M, Schliebs R, Rossner S, Bigl V (1997). Basal forebrain cholinergic immunolesion by 192lgG-saporin: evidence for a presynaptic location of subpopulations of alpha 2- and beta-adrenergic as well as 5- HT2A receptors on cortical cholinergic terminals. Neurochem. Res.

[171] Heishman SJ, Henningfield JE (1991). Discriminative stimulus effects of d-amphetamine, methylphenidate, and diazepam in humans. Psychopharmacology (Berl.).

[172] Heneka MT, Ramanathan M, Jacobs AH, Dumitrescu-Ozimek L, Bilkei-Gorzo A, Debeir T, Sastre M, Galldiks N, Zimmer A, Hoehn M, Heiss W-D, Klockgether T, Staufenbiel M (2006). Locus coeruleus degeneration promotes Alzheimer pathogenesis in amyloid precursor protein 23 transgenic mice. J. Neurosci.

[173] Herman JP, Ostrander MM, Mueller NK, Figueiredo H (2005). Limbic system mechanisms of stress regulation: hypothalamo-pituitary- adrenocortical axis. Prog. Neuropsychopharmacol. Biol. Psychiatry.

[174] Hermann DM, Luppi P-H, Peyron C, Hinckel P, Jouvet M (1997). Afferent projections to the rat nuclei raphe magnus, raphe pallidus and reticularis gigantocellularis pars α demonstrated by iontophoretic application of choleratoxin (subunit b). J. Chem. Neuroanat.

[175] Hey JA, Gherezghiher T, Koss MC (1985). Studies on the mechanism of clonidine-induced mydriasis in the rat. Naunyn-Schmiedebergs Arch. Pharmacol.

[176] Hey JA, Ito T, Koss MC (1989). Mechanism of dexamphetamine- induced mydriasis in the anaesthetised rat. Br. J. Pharmacol.

[177] Hey JA, Koss MC (1988). Alpha_1_- and alpha_2_-adrenoreceptor antagonists produce opposing mydriatic effects by a central action. J. Auton. Pharmacol.

[178] Higgins ST, Rush CR, Bickel WK, Hughes JR, Lynn M, Capeless MA (1993). Acute behavioural and cardiac effects of cocaine and alcohol combinations in humans. Psychopharmacology (Berl.).

[179] Hill JL, Zacny JP (2000). Comparing the subjective, psychomotor, and physiological effects of intravenous hydromorphone and morphine in healthy volunteers. Psychopharmacology (Berl.).

[180] Hoefke VW, Kobinger W (1966). Pharmakologische Wirkungen des 2- (2,6-dichlorphenylamino)-2-imidazolin-hydrochlorids, einer neuen, antihypertensive substanz. Arneimittelforschung.

[181] Hoogendijk WJ, Feenstra MG, Botterblom MH, Gilhuis J, Sommer IE, Kamphorst W, Eikelenbool P, Swaab DF (1999). Increased activity of surviving locus coeruleus neurons in Alzheimer’s disease. Ann. Neurol.

[182] Hossmann V, Maling TJ, Hamilton CA, Reid JL, Dollery CT (1980). Sedative and cardiovascular effects of clonidine and nitrazepam. Clin. Pharmacol. Ther.

[183] Hotson JR, Steinke GW (1988). Vertical and horizontal saccades in aging and dementia. Neuro ophthalmology.

[184] Hou RH, Freeman C, Langley RW, Szabadi E, Bradshaw CM (2005). Does modafinil activate the locus coeruleus in man? Comparison of modafinil and clonidine on arousal and autonomic functions in human volunteers. Psychopharmacology (Berl.).

[185] Hou YP, Manns ID, Jones B (2002). Immunostaining of cholinergic pontomesencephalic neurons for α1 versus α2 adrenergic receptors suggests different sleep-wake state activities and roles. Neuroscience.

[186] Hou RH, Scaife J, Freeman C, Langley RW, Szabadi E, Bradshaw CM (2006). Relationship between sedation and pupillary function: comparison of diazepam and diphenhydramine. Br. J. Clin. Pharmacol.

[187] Hou RH, Langley RW, Szabadi E, Bradshaw CM (2007). Comparison of diphenhydramine and modafinil on arousal and autonomic functions in healthy volunteers. J. Psychopharmacol.

[188] Hou RH, Samuels ER, Raisi M, Langley RW, Szabadi E, Bradshaw CM (2006). Why patients with Alzheimer’s disease may show increased sensitivity to tropicamide eye drops: Role of locus coeruleus. Psychopharmcology.

[189] Hou RH, Samuels ER, Langley RW, Szabadi E, Bradshaw CM (2007). Arousal and the pupil: Why diazepam-induced sedation is not accompanied by miosis. Psychopharmacology (Berl.).

[190] Huang Z-L, Urade Y, Hayaishi O (2007). Prostaglandins and adenosine in the regulation of sleep and wakefulness. Curr. Opin. Pharmacol.

[191] Hutton JT, Nagel JA, Loewenson RB (1984). Eye tracking dysfunction in Alzheimer-type dementia. Neurology.

[192] Hwang K-R, Chan SHH, Chan JYH (1998). Noradrenergic neurotransmission at PVN in locus coeruleus-induced baroreflex suppression in rats. Heart Circ. Physiol.

[193] Ida Y, Tanaka M, Tsuda A, Tsujimaru S, Nagasaki N (1985). Attenuating effect of diazepam on stress-induced increases in noradrenaline turnover in specific brain regions of rats: antagonism by Ro 15-1788. Life Sci.

[194] Ilbäck N-G, Siller M, Stålhandsje T (2007). Evaluation of cardiovascular effects of caffeine using telemetric monitoring in the conscious rat. Food Chem. Toxicol.

[195] Ishida Y, Hashiguchi H, Takeda R, Ishizuka Y, Mitsuyama Y, Kannan H, Nishimori T, Nakahara D (2002). Conditioned-fear stress increases fos expression in monoaminergic and GABAergic neurons of the locus coeruleus and dorsal raphe nuclei. Synapse.

[196] Ishida Y, Shirokawa T, Komatsu Y, Isobe K (2001). Changes in cortical noradrenergic axon terminals of locus coeruleus neurons in aged F344 rats. Neurosci. Lett.

[197] Ishida Y, Shirokawa T, Miyaishi O, Komatsu Y, Isobe K (2000). Age- dependent changes in projections from locus coeruleus to hippocampus dentate gyrus and frontal cortex. Eur. J. Neurosci.

[198] Ishida Y, Shirokawa T, Miyaishi O, Komatsu Y, Isobe K (2001). Age-dependent changes in noradrenergic innervations of the frontal cortex in F344 rats. Neurobiol. Aging.

[199] Ivanenko A, Tauman R, Gozal D (2003). Modafinil in the treatment of excessive daytime sleepiness in children. Sleep Med.

[200] Ivanov A, Aston-Jones G (1995). Extranuclear dendrites of locus coeruleus neurons: activation by glutamate and modulation of activity by alpha adrenoceptors. J. Neurophysiol.

[201] Iversen LL, Rossor MN, Reynolds GP, Hills R, Roth M, Mountjoy CQ, Foote SL, Morrison JH, Bloom FE (1983). Loss of pigmented dopamine-β-hydroxylase positive cells from locus coeruleus in senile dementia of Alzheimer’s type. Neurosci. Lett.

[202] Jaanus SD (1992). Ocular side effects of selected systemic drugs. Optom. Clin.

[203] Jansen AS, Ter Horst GJ, Mettenleiter TC, Loewy AD (1992). CNS cell groups projecting to the submandibular parasympathetic preganglionic neurons in the rat: a retrograde transneuronal viral cell body labeling study. Brain Res.

[204] Johnson MA, Blackwell CP, Smith J (1995). Antagonism of the effects of clonidine by the α_2_-adrenoceptor antagonist, fluparoxan. Br. J. Clin. Pharmacol.

[205] Jones BE (2004). Activity, modulation and role of basal forebrain cholinergic neurons innervating the cerebral cortex. Prog. Brain Res.

[206] Jones BE (2005). From waking to sleeping: neuronal and chemical substrates. Trends Pharmacol. Sci.

[207] Jones BE, Moore RY (1977). Ascending projections of the locus coeruleus in the rat. II Autoradiographic study. Brain Res.

[208] Jones BE, Yang T-Z (1985). The efferent projections from the reticular formation and the locus coeruleus studies by anterograde and retrograde axonal transport in the rat. J. Comp. Neurol.

[209] Jorm CM, Stamford JA (1993). Actions of the hypnotic anaesthetic, dexmedetomidine, on noradrenaline release and cell firing in rat locus coeruleus slices. Br. J. Anaesth.

[210] Jovanovic T, Norrholm SD, Fiallos A, Myers KM, Davis M, Keyes M, Jovanovic S, Duncan EJ (2006). Contingency awareness and fear inhibition in a human fear-potentiated startle paradigm. Behav. Neurosci.

[211] Kaitin KI, Bliwise DL, Gleason C, Nino-Murcia G, Dement WC, Libet B (1986). Sleep disturbance produced by electrical stimulation of the locus coeruleus in a human subject. Biol. Psychiatry.

[212] Kalsbeek A, Garidou ML, Palm IF, Van Der Vliet J, Simonneaux V, Pevet P, Buijs RM (2000). Melatonin sees the light: blocking GABA- ergic transmission in the paraventricular nucleus induces daytime secretion of melatonin. Eur. J. Neurosci.

[213] Kamimori GH, Penetar DM, Headley DB, Thorne DR, Otterstetter R, Belenky G (2000). Effect of three caffeine doses on plasma catecholamines and alertness during prolonged wakefulness. Eur. J. Clin. Pharmacol.

[214] Kaniucki MD, Stefano FJ, Perec CJ (1984). Clonidine inhibits salivary secretion by activation of postsynaptic alpha 2-receptors. Naunyn- Schmiedebergs Arch. Pharmacol.

[215] Katz RJ (1979). Opiate stimulation increases exploration in the mouse. Int. J. Neurosci.

[216] Kaur S, Saxena RN, Mallick BN (1997). GABA in locus coeruleus regulates spontaneous rapid eye movement sleep by acting on GABAA receptors in freely moving rats. Neurosci. Lett.

[217] Keating GL, Rye DB (2003). Where you least expect it: dopamine in the pons and modulation of sleep and REM-sleep. Sleep.

[218] Kiernan JA (2005). Barr’s the human nervous system: an anatomical viewpoint.

[219] Kim M-A, Lee HS, Lee BY, Waterhouse BD (2004). Reciprocal connections between subdivisions of the dorsal raphe and the nuclear core of the locus coeruleus in the rat. Brain Res.

[220] Kimura F, Nakamura S (1985). Locus coeruleus neurons in the neonatal rat: electrical activity and responses to sensory stimulation. Brain Res.

[221] King C, Masserano JM, Codd E, Byrne WL (1981). Effects of beta- endorphin and morphine on the sleep-wakefulness behavior of cats. Sleep.

[222] King DJ, Waddington JL, King DJ (2004). Antipsychotic drugs and treatment of schizophrenia. Seminars in Clinical Psychopharmacology.

[223] Kiyohara T, Miyata S, Nakamura T, Shido O, Nakashima T, Shibata M (1995). Differences in Fos expression in the rat brains between cold and warm ambient exposures. Brain Res. Bull.

[224] Klemfuss H, Adler MW (1986). Autonomic mechanisms for morphine and amphetamine mydriasis in the rat. J. Pharmacol. Exp. Ther.

[225] Knaggs RD, Crighton IM, Cobby TF, Fletcher AJP, Hobbs GJ (2004). The pupillary effects of intravenous morphine, codeine, and tramadol in volunteers. Anesth. Analg.

[226] Koch M (1999). The neurobiology of startle. Prog. Neurobiol.

[227] Kocsis B, Li S, Hajos M (2007). Behavior-dependent modulation of hippocampal EEG activity by the selective norepinephrine reuptake inhibitor reboxetine in rats. Hippocampus.

[228] Koob GF, Maldonado R, Stimus L (1992). Neural substrates of opiate withdrawal. Trends Neurosci.

[229] Koot P, Deurenberg P (1995). Comparison of changes in energy expenditure and body temperatures after caffeine consumption. Ann. Nutr. Metab.

[230] Korczyn AD, Maor D (1982). Central and peripheral components of morphine mydriasis in mice. Pharmacol. Biochem. Behav.

[231] Koss MC (1986). Pupillary dilation as an index of central nervous system α_2_-adrenoceptor activation. J. Pharmacol. Methods.

[232] Koss MC, San LC (1976). Analysis of clonidine-induced mydriasis. Invest. Opthalmol.

[233] Kumari V, Cotter P, Corr PJ, Gray JA, Checkley SA (1996). Effect of clonidine on the human acoustic startle reflex. Psychopharmacology (Berl.).

[234] Kuskowski MA, Malone SM, Mortimer JA, Dysken MW (1989). Smooth pursuit eye movements in dementia of the Alzheimer’s type. Alzheimer Dis. Assoc. Disord.

[235] Lamb K, Bradshaw CM, Szabadi E (1983). The responsiveness of human eccrine sweat glands to choline and carbachol. Eur. J. Clin. Pharmacol.

[236] Lane-Ladd SB, Pineda J, Boundy VA, Pfeuffer T, Krupinski J, Aghajanian GK, Nestler EJ (1997). CREB (cAMP response element- binding protein) in the locus coeruleus: biochemical, physiological, and behavioural evidence for a role in opiate dependence. J. Neurosci.

[237] Larson MD, Talke PO (2001). Effect of dexmedetomidine, an α_2_- adrenoceptor agonist, on human pupillary reflexes during general anaesthesia. Br. J. Clin. Pharmacol.

[238] Laubie M, Schmitt H (1977). Sites of action of clonidine: centrally mediated increase in vagal tone, centrally mediated hypotensive and sympatho-inhibitory effects. Hypertens. Brain Mech.

[239] Laurent JP, Mangold M, Humbel U, Haefely W (1983). Reduction by two benzodiazepines and pentobarbitone of the multiunit activity in substantia nigra, hippocampus, nucleus locus coeruleus and nucleus raphe dorsalis of encephale isole rats. Neuropharmacology.

[240] Laurie DJ, Pratt JA (1989). Local cerebral glucose utilization following subacute and chronic diazepam pre-treatment: differential tolerance. Brain Res.

[241] Laverty R, Taylor KM (1969). Behavioural and biochemical effects of 2- (2,6-dichlorophenylamino)-2-imidazoline hydrochloride (ST 155) on the central nervous system. Br. J. Pharmacol.

[242] LeDoux J (1998). Fear and the brain: where have we been, and where are we going?. Biol. Psychiatr.

[243] Lee CR, McTavish D, Sorkin EM (1993). Tramadol. A preliminary review of its pharmacodynamic and pharmacokinetic properties, and therapeutic potential in acute and chronic pain states. Drugs.

[244] Lees AJ (1993). Dopamine agonists in Parkinson's disease : a look at apomorphine. Fund. Clin. Pharmacol.

[245] Leppävuori A, Putkonen PT (1980). Alpha-adrenoceptive influences on the control of the sleep-waking cycle in the cat. Brain Res.

[246] Leslie FM, Loughlin SE, Sternberg DB, McGaugh JL, Young LE, Zornetzer SF (1985). Noradrenergic changes and memory loss in aged mice. Brain Res.

[247] Leung NK, Bradshaw CM, Szabadi E (1992). Effect of high ambient temperature on the kinetics of the pupillary light reflex in healthy volunteers. Br. J. Clin. Pharmacol.

[248] Lewis DI, Coote JH (1990). Excitation and inhibition of rat sympathetic preganglionic neurones by catecholamines. Brain Res.

[249] Lewis KS, Han NH (1997). Tramadol: a new centrally acting analgesic. Am. J. Health Syst. Pharm.

[250] Liddell BJ, Brown KJ, Kemp AH, Barton MJ, Das P, Peduto A, Gordon E, Williams LM (2005). A direct brainstem-amygdala-cortical “alarm” system for subliminal signals of fear. NeuroImage.

[251] Lightman SL, Todd K, Everitt BJ (1984). Ascending noradrenergic projections from the brainstem: evidence for a major role in the regulation of blood pressure and vasopressin secretion. Exp. Brain Res.

[252] Lipski JR, Kanjhan B, Krusezeska Smith M (1995). Barosensitive neurones in rostral ventrolateral medulla of a rat *in vivo*: morphological properties and relationship to C1 adrenergic neurons. Neuroscience.

[253] Liu X, Tang X, Sanford LD (2003). Fear-conditioned suppression of REM sleep: relationship to fos expression patterns in limbic and brainstem regions in BALB/cJ mice. Brain Res.

[254] Loewenfeld IE (1993). The Pupil: anatomy, physiology, and clinical applications.

[255] Loewy AD, Araujo JC, Kerr FWL (1973). Pupillodilator pathways in the brain stem of the cat: anatomical and electrophysiological identification of a central autonomic pathway. Brain Res.

[256] Loewy AD, Spyer KM, Loewy AD, Spyer KM (1990). Vagal preganglionic neurons. Oxford Central Regulation of Autonomic Functions.

[257] Lowenstein O, Feinbeig R, Loewenfeld IE (1963). Pupillary movements during acute and chronic fatigue. Invest. Opthalmol.

[258] Lu J, Jhou TC, Saper CB (2006). Identification of wake-active dopaminergic neurons in the ventral periaqueductal gray matter. J. Neurosci.

[259] Lyness SA, Zarow C, Chui HC (2003). Neuron loss in key cholinergic and aminergic nuclei in Alzheimer disease: a meta-analysis. Neurobiol. Aging.

[260] MacDonald JR, Hill JD, Tarnopolsky MA (2002). Modafinil reduces excessive somnolence and enhances mood in patients with myotonic dystrophy. Neurology.

[261] MacDougall AI, Addis GJ, MacKay N, Dymock IW, Turpie AGG, Ballingall DLK, MacLennan WJ, Whiting B, MacArthur JG (1970). Treatment of hypertension with clonidine. Br. Med. J.

[262] Madras BK, Xie Z, Lin Z, Jassen A, Panas H, Lynch L, Johnson R, Livni E, Spencer TJ, Bonab AA, Miller GM, Fischman AJ (2006). Modafinil occupies dopamine and norepinephrine transporters *in vivo* and modulates the transporters and trace amine activity *in vitro*. J. Pharmacol. Exp. Ther.

[263] Maeda T, Kitahama K, Geffard M (1994). Dopaminergic innervation of rat locus coeruleus: a light and electron microscope immunohistochemical study. Microsc. Res. Tech.

[264] Makris AP, Rush CR, Frederich RC, Kelly TH (2004). Wake- promoting agents with different mechanisms of action: comparison of effects of modafinil and amphetamine on food intake and cardiovascular activity. Appetite.

[265] Manaye KF, McIntire DD, Mann DMA, German DC (1995). Locus coeruleus cell loss in the aging human brain: a non-random process. J. Comp. Neurol.

[266] Mann DM, Yates PO, Hawkes J (1983). The pathology of the human locus coeruleus. Clin. Neuropathol.

[267] Mann DM, Yates PO, Marcyniuk B (1984). A comparison of changes in the nucleus basalis and locus coeruleus in Alzheimer’s disease. J. Neurol. Neurosurg. Psychiatry.

[268] Manns ID, Lee MG, Modirrousta M, Hou YP, Jones BE (2003). Alpha 2 adrenergic receptors on GABA-ergic, putative sleep-promoting basal forebrain neurons. Eur. J. Neurosci.

[269] Mantz J (1999). Dexmedetomidine. Drugs Today (Barc.).

[270] Marcyniuk B, Mann DM, Yates PO (1989). The topography of nerve cell loss from the locus coeruleus in elderly persons. Neurobiol. Aging.

[271] Marcyniuk B, Mann DM, Yates PO, Ravindra CR (1988). Topography of nerve cell loss from the locus coeruleus in middle aged persons with Down’s syndrome. J. Neurol. Sci.

[272] Marien MR, Colpaert FC, Rosenquist AC (2004). Noradrenergic mechanisms in neurodegenerative diseases: a theory. Brain Res. Rev.

[273] Marwaha J, Kehne JH, Commissaris RL, Lakoski J, Shaw W, Davis M (1983). Spinal clonidine inhibits neural firing in locus coeruleus. Brain Res.

[274] Masson J, Sagn C, Hamon M, Mestikawy SE (1999). Neurotransmitter transporters in the central nervous system. Pharmacol. Rev.

[275] Matthews KL, Chen CPL-H, Esiri MM, Keene J, Minger SL, Francis PT (2002). Noradrenergic changes, aggressive behavior, and cognition in patients with dementia. Biol. Psychiatry.

[276] Mavridis M, Degryse AD, Lategan AJ, Marien MR, Colpaert FC (1991). Effects of locus coeruleus lesions on parkinsonian signs, striatal dopamine and substantia nigra cell loss after 1-methyl-4-phenyl-1,2,3,6- tetrahydropyridine in monkeys: a possible role for the locus coeruleus in the progression of Parkinson’s disease. Neuroscience.

[277] Max MB, Schafer SC, Culnane M, Dubner R, Gracely RH (1988). Association of pain relief with drug side effects in postherpetic neuralgia: a single-dose study of clonidine, codeine, ibuprofen, and placebo. Clin. Pharmacol. Ther.

[278] May CN, Ham IW, Heslop KE, Stone FA, Mathias CJ (1988). Intravenous morphine causes hypertension, hyperglycaemia and increases sympatho-adrenal outflow in conscious rabbits. Clin. Sci. (Lond.).

[279] Mayer AF, Schroeder C, Heusser K, Tank J, Diedrich A, Schmieder RE, Luft FC, Jordan J (2006). Influences of norepinephrine transporter function on the distribution of sympathetic activity in humans. Hypertension.

[280] McCormick DA, Bal T (1997). Sleep and arousal: thalamocortical mechanisms. Ann. Rev. Neurosci.

[281] McCormick DA, Pape HC, Williamson A (1991). Actions of norepinephrine in the cerebral cortex and thalamus: implications for function of the central noradrenergic system. Prog. Brain Res.

[282] McDougle CJ, Krystal JH, Price LH, Heninger GR, Charney DS (1995). Noradrenergic response to acute ethanol administration in healthy subjects: comparison with intravenous yohimbine. Psychopharmacology (Berl.).

[283] McLellan TM, Ducharme MB, Canini F, Moroz D, Bell DG, Baranski JV, Gil V, Buguet A, Radomski MW (2002). Effect of modafinil on core temperature during sustained wakefulness and exercise in a warm environment. Aviat. Space Environ. Med.

[284] Mehta MC, Jain AC, Billie M (2004). Effects of cocaine and caffeine alone and in combination on cardiovascular performance. An experimental hemodynamic and coronary flow reserve study in a canine model. Int. J. Cardiol.

[285] Merrill CA, Jonsson MAG, Minthon L, Ejnell H, Silander HC, Blennow K, Karlsson M, Nordlund A, Rolstad S, Warkentin S, Ben- Menachem E, Sjögren MJC (2006). Vagus nerve stimulation in patients with Alzheimer’s disease: additional follow-up results of a pilot study through 1 year. J. Clin. Psychiatry.

[286] Miller CD, Asbury AJ, Brown JH (1990). Pupillary effects of alfentanil and morphine. Br. J. Anaesth.

[287] Mitler MM, O'Malley MB, Kryger MH, Roth T, Dement WC (2005). Wake-promoting medications: efficacy and adverse effects. Principles and Practice of Sleep Medicine.

[288] Modirrousta M, Mainville L, Jones BE (2004). GABAergic neurons with α_2_-adrenergic receptors in basal forebrain and preoptic area express c-Fos during sleep. Neuroscience.

[289] Mohler H, Fritschy JM, Rudolph U (2002). A new benzodiazepine pharmacology. J. Pharmacol. Exp. Ther.

[290] Molderings GJ, Bönisch H, Bruss M, Göthert M (2003). Alpha_2A_- adrenergic versus imidazoline receptor controversy in rilmenidine's action: alpha_2A_-antagonism in humans versus alpha_2A_-agonism in rabbitsrabbits. In: Agmatine and Imidazolines: Their Novel Receptors and Enzymes. Ann. N.Y. Acad. Sci..

[291] Möller H-J (2003). Amisulpride: limbic specificity and the mechanism of antipsychotic atypicality. Prog. Neuropsychopharmacol. Biol. Psychiatry.

[292] Montastruc P, Berlan M, Montastruc JL (1989). Effects of yohimbine on submaxillary salivation in dogs. Br. J. Pharmacol.

[293] Moore RY, Bloom FE (1979). Central catecholamine neuron systems: anatomy and physiology of the norepinephrine and epinephrine systems. Ann. Rev. Neurosci.

[294] Morgan CA, Southwick SM, Grillon C, Davis M, Krystal JH, Charney DS (1993). Yohimbine-facilitated acoustic startle reflex in humans. Psychopharmacology (Berl.).

[295] Morilak DA, Fornal CA, Jacobs BL (1987). Effects of physiological manipulations on locus coeruleus neuronal activity in freely moving cats I. Thermoregulatory challenge. Brain Res.

[296] Morley MJ, Bradshaw CM, Szabadi E (1991). Effects of clonidine and yohimbine on the pupillary light reflex and carbachol-evoked sweating in healthy volunteers. Br. J. Clin. Pharmacol.

[297] Murakami S, Okamura H, Yanaihara C, Yanaihara N, Ibata Y (1987). Immunocytochemical distribution of met-enkephalin-Arg^6^-Gly^7^-Leu^8^ in the rat lower brainstem. J. Comp. Neurol.

[298] Murillo-Rodríguez E, Haro R, Palomero-Rivero M, Millán-Aldaco D, Drucker-Colín R (2007). Modafinil excites extracellular levels of dopamine in the nucleus accumbens and increases wakefulness in rats. Behav. Brain Res.

[299] Muzi M, Goff DR, Kampine JP, Roerig DL, Ebert TJ (1992). Clonidine reduces sympathetic activity but maintains baroreflex responses in normotensive humans. Anesthesiology.

[300] Myers EA, Banihashemi L, Rinaman L (2005). The anxiogenic drug yohimbine activates central viscerosensory circuits in rats. J. Comp. Neurol.

[301] Myers K, Goulet M, Rusche J, Boismenu R, Davis M (2004). Inhibition of fear potentiated startle in rats following peripheral administration of secretin. Psychopharmacology (Berl.).

[302] Nahas Z, Marangell LB, Husain MM, Rush AJ, Sackeim HA, Lisanby SH, Martinez JM, George MS (2005). Two-year outcome of vagus nerve stimulation (VNS) for treatment of major depressive episodes. J. Clin. Psychiatry.

[303] Nakai T, Hayashi M, Ichihara K, Wakabayashi H, Hoshi K (2002). Noradrenaline release in rat locus coeruleus is regulated by both opioid and α_2_-adenoceptors. Pharmacol. Res.

[304] Nelson LE, Guo TZ, Lu J, Saper CB, Franks NP, Maze M (2002). The sedative component of anesthesia is mediated by GABA_A_ receptors in an endogenous sleep pathway. Nat. Neurosci.

[305] Nelson LE, Lu J, Guo TZ, Saper CB, Franks NP, Maze M (2003). The α2-adrenoceptor agonist dexmedetomidine converges on an endogenous sleep-promoting pathway to exert its sedative effects. Anesthesiology.

[306] Neophytou SI, Aspley S, Butler S, Beckett S, Marsden CA (2001). Effects of lesioning noradrenergic neurones in the locus coeruleus onconditioned and unconditioned aversive behaviour in the rat. Prog. Neuro Psychopharmacol. Biol. Psychiatry.

[307] Newton TF, De La Garza R, Kalechstein AD, Nestor L (2005). Cocaine and methamphetamine produce different patterns of subjective and cardiovascular effects. Pharmacol. Biochem. Behav.

[308] Niederhoffer N, Hein L, Starke K (2004). Modulation of the baroreceptor reflex by α_2A_-adrenoceptors: a study in α_2A_ knockout mice. Br. J. Pharmacol.

[309] Nieuwenhuys R (1985). Chemoarchitecture of the brain.

[310] Nieves AV, Lang AE (2002). Treatment of excessive daytime sleepiness in patients with Parkinson’s disease with modafinil. Clin. Neuropharmacol.

[311] Nishino S, Mao J, Sampathkumaran R, Shelton J, Mignot E (1998). Increased dopaminergic transmission mediates the wake-promoting effects of CNS stimulants. Sleep Res. Online.

[312] Nistico G, De Sarro GB (1990). Altered responsiveness of central alpha 2- adrenoceptors in aging. Ann. Inst. Super. Sanita.

[313] Nistico G, De Sarro GB, Bagetta G, Mollace V (1992). Altered sensitivity of alpha 2-adrenoceptors in the brain during aging in rats. Ann. N. Y. Acad. Sci.

[314] Nitz D, Siegel JM (1997). GABA release in the locus coeruleus as a function of sleep/wake state. Neuroscience.

[315] Noordzij M, Uiterwaal CS, Arends LR, Kok FJ, Grobbee DE, Geleijnse JM (2005). Blood pressure response to chronic intake of coffee and caffeine: a meta-analysis of randomized controlled trials. J. Hypertens.

[316] Nygren L-G, Olson L (1977). A new major projection from locus coeruleus: the main source of noradrenergic nerve terminals in the ventral and dorsal columns of the spinal cord. Brain Res.

[317] Ornstein K, Milon H, McRae-Degueurce A, Alvarez C, Berger B, Würzner HP (1987). Biochemical and radioautographic evidence for dopaminergic afferents of the locus coeruleus originating in the ventral tegmental area. J. Neural Transm.

[318] Orrell RW, King AW, Hilton DA, Campbell MJ, Lane RJ, de Belleroche JS (1995). Familial amyotrophic lateral sclerosis with a point mutation of SOD-1: intrafamilial heterogeneity of disease duration associated with neurofibrillary tangles. J. Neurol. Neurosurg. Psychiatry.

[319] Osaka T, Matsumura H (1994). Noradrenergic inputs to sleep-related neurons in the preoptic area from the locus coeruleus and the ventrolateral medulla in the rat. Neurosci. Res.

[320] Osterhout CA, Sterling CR, Chikaraishi DM, Tank AW (2005). Induction of tyrosine hydroxylase in the locus coeruleus of transgenic mice in response to stress or nicotine treatment: lack of activation of tyrosine hydroxylase promoter activity. J. Neurochem.

[321] Pacak K, Armando I, Fukuhara K, Kvetnansky R, Palkovits M, Kopin IJ, Goldstein DS (1992). Noradrenergic activation in the paraventricular nucleus during acute and chronic immobilization stress in rats: an *in vivo* microdialysis study. Brain Res.

[322] Pacak K, Palkovits M, Kvetnansky R, Fukuhara K, Armando I, Kopin IJ, Goldstein DS (1993). Effects of single or repeated immobolization on release of norepinephrine and its metabolites in the central nucleus of the amygdala in conscious rats. Neuroendocrinology.

[323] Pacak K, Palkovits M, Kopin IJ, Goldstein DS (1995). Stress induced norepinephrine release in the hypothalamic paraventricular nucleus and pituitary-adrenocortical and sympathoadrenal activity: *in vivo* micodialysis studies. Front. Neuroendocrinol.

[324] Pacák K, Palkovits M (2001). Stressor specificity of central neuroendocrine responses: implications for stress-related disorders. Endocr. Rev.

[325] Pack AI, Black JE, Schwartz JRL, Matheson JK (2001). Modafinil as adjunct therapy for daytime sleepiness in obstructive sleep apnea. Am. J.Respir. Crit. Care Med.

[326] Palkovits M, Baffi JS, Dvori S (1995). Neuronal organisation of stress response. Pain-induced c-fos expression in brain stem catecholaminergic cell groups. Ann. N. Y. Acad. Sci.

[327] Palkovits M, Baffi JS, Pacák K (1997). Stress-induced Fos-like immunoreactivity in the pons and the medulla oblongata of rats. Stress.

[328] Palkovits M, Baffi JS, Pacák K (1999). The role of ascending neuronal pathways in stress-induced release of noradrenaline in the hypothalamic paraventricular nucleus of rats. J Neuroendocrinol.

[329] Parini A, Moudanos CG, Pizzinat N, Lanier SM (1996). The elusive family of imidazoline binding sites. Trends Pharmacol. Sci.

[330] Parvizi J, Damasio AR (2003). Neurochemical correlates of brainstem coma. Brain.

[331] Patat A, Rosenzweig P, Miget N, Allain H, Gandon J-M (1999). Effects of 50mg amisulpride on EEG, psychomotor and cognitive functions in healthy sleep-deprived subjects. Fundam. Clin. Pharmacol.

[332] Patt S, Gerhard L (1993). A golgi study of human locus coeruleus in normal brains and in Parkinson’s disease. Neuropathol. Appl. Neurobiol.

[333] Paus S, Brecht HM, Koster J, Seeger G, Klockgether T, Wullner U (2003). Sleep attacks, daytime sleepiness, and dopamine agonists in Parkinson’s disease. Mov. Disord.

[334] Peacock JE, Henderson PD, Nimmo WS (1988). Changes in pupil diameter after oral administration of codeine. Br. J. Anaesth.

[335] Penetar D, McCann U, Thorne D, Kamimori G, Galinski C, Sing H, Thomas M, Belenky G (1993). Caffeine reversal of sleep deprivation effects on alertness and mood. Psychopharmacology (Berl.).

[336] Penttila J, Helminen A, Anttila M, Hinkka S, Scheinin H (2004). Cardiovascular and parasympathetic effects of dexmedetomidine in healthy subjects. Can. J. Physiol. Pharmacol.

[337] Perrault GH, Depoortere R, Morel E, Sanger DJ, Scatton B (1997). Psychopharmacological profile of amisulpride: an antipsychotic drug with presynaptic D2/D3 dopamine receptor antagonist activity and limbic selectivity. J. Pharmacol. Exp. Ther.

[338] Pérez MF, Nasif FJ, Marchesini GR, Maglio LE, Ramirez OA (2001). Hippocampus and locus coeruleus activity on rats chronically treated with diazepam. Pharmacol. Biochem. Behav.

[339] Perl DP, Olanow CW, Calne D (1998). Alzheimer’s disease and Parkinson’s disease: distinct entities or extremes of a spectrum of neurodegeneration. Ann. Neurol.

[340] Pezzone MA, Lee W-S, Hoffman GE, Pezzone KM, Rabin BS (1993). Activation of brainstem catecholaminergic neurons by conditioned and unconditioned aversive stimuli as revealed by c-Fos immunoreactivity. Brain Res.

[341] Phillips MA, Szabadi E, Bradshaw CM (2000). Comparison of the effects of clonidine and yohimbine on pupillary diameter at different illumination levels. Br. J. Clin. Pharmacol.

[342] Phillips MA, Szabadi E, Bradshaw CM (2000). Comparison of the effects of clonidine and yohimbine on spontaneous pupillary fluctuations in healthy human volunteers. Psychopharmacology (Berl.).

[343] Pickworth WB, Bunker E, Welch P, Cone E (1991). Intravenous buprenorphine reduces pupil size and the light reflex in humans. Life Sci.

[344] Pickworth WB, Lee H, Fudala PJ (1990). Buprenorphine-induced pupillary effects in human volunteers. Life Sci.

[345] Pickworth WB, Welch P, Henningfield JE, Cone EJ (1989). Opiate- induced pupillary effects in humans. Methods Find. Exp. Clin. Pharmacol.

[346] Pigeau R, Naitoh P, Buguet A, McCann C, Baranski J, Taylor M, Thompson M, Mack II (1995). Modafinil, d-amphetamine and placebo during 64 hours of sustained mental work. I. Effects on mood, fatigue, cognitive performance and body temperature. J. Sleep Res.

[347] Pirker S, Schwarzer C, Wieselthaler A, Sieghart W, Sperk G (2000). GABA(A) receptors: immunocytochemical distribution of 13 subunits in the adult rat brain. Neuroscience.

[348] Pitts DK, Marwah J (1986). Effects of cocaine on the electrical activity of single noradrenergic neurons from locus coeruleus. Life Sci.

[349] Pitts DK, Marwah J (1987). Reciprocal pre- and postsynaptic actions of cocaine at a central noradrenergic synapse. Exp. Neurol.

[350] Pitts DK, Marwah J (1987). Electrophysiological actions of cocaine on noradrenergic neurons in rat locus coeruleus. J. Pharmacol. Exp. Ther.

[351] Pitts DK, Marwah J (1988). Cocaine-elicited mydriasis in the rat: pharmacological comparison to clonidine, D-amphetamine and desipramine. J. Pharmacol. Exp. Ther.

[352] Pozzessere G, Valle E, Rossi P, Petrucci B, Ambrosini A, D'Alessio M, Pierelli F, Giacomini P (1996). Pupillometric evaluation and analysis of light reflex in healthy subjects as a tool to study autonomic nervous system changes with aging. Aging (Milano).

[353] Prettyman R, Bitsios P, Szabadi E (1997). Altered pupillary size and darkness and light reflexes in Alzheimer’s disease. J. Neurol. Neurosurg. Psychiatry.

[354] Rajkowski J, Kubiak P, Aston-Jones G (1993). Correlations between locus coeruleus (LC) neural activity, pupil diameter and behaviour in monkey support a role of LC in attention. Abstr. Soc. Neurosci.

[355] Rajkowski J, Kubiak P, Aston-Jones G (1994). Locus coeruleus activity in monkey: phasic and tonic changes are associated with altered vigilance. Brain Res. Bull.

[356] Rajput AH, Uitti RJ, Sudhakar S, Rozdilsky B (1989). Parkinsonism and neurofibrillary tangle pathology in pigmented nuclei. Ann. Neurol.

[357] Ramesh V, Kumar VM (1998). The role of alpha-2 receptors in the medial preoptic area in the regulation of sleep-wakefulness and body temperature. Neuroscience.

[358] Rammohan KW, Rosenberg JH, Lynn DJ, Blumenfeld AM, Pollak CP, Nagaraja HN (2002). Efficacy and safety of modafinil (Provigil) for the treatment of fatigue in multiple sclerosis: a two centre phase 2 study. J. Neurol. Neurosurg. Psychiatry.

[359] Rasmussen K, Aghajanian GK (1990). Serotonin excitation of facial motoneurons: receptor subtype characterization. Synapse.

[360] Rasmussen K, Jacobs BL (1986). Single unit activity of locus coeruleus neurons in the freely moving cat. II. Conditioning and pharmacologic studies. Brain Res.

[361] Rasmussen K, Morilak DA, Jacobs BL (1986). Single unit activity of locus coeruleus neurons in the freely moving cat. I. During naturalistic behaviors and in response to simple and complex stimuli. Brain Res.

[362] Ravanelli MIB, Almeida MC, Branco LGS (2007). Role of the locus coeruleus carbon monoxide pathway in endotoxin fever in rats. Pflugers Arch. Eur. J. Physiol.

[363] Redmond DE, Meltzer HY (1987). Studies of the nucleus locus coeruleus in monkeys and hypotheses for neuropsychopharmacology. Psychopharmacology: The Third Generation of Progress.

[364] Redmond DE, Huang YH (1979). New evidence for a locus coeruleus- norepinephrine connection with anxiety. Life Sci.

[365] Redmond DE, Huang YH, Snyder DR, Maas JW (1976). Behavioral effects of stimulation of the locus coeruleus in the stumptail monkey (*macaca arctoides*). Brain Res.

[366] Reid JL, Lewis PJ, Meyers MG (1975). Role of central dopaminergic mechanisms in piribedil and clonidine induced hypothermia in the rat. Neuropharmacology.

[367] Remy P, Doder M, Lees A, Turjanski N, Brooks D (2005). Depression in Parkinson’s disease: loss of dopamine and noradrenaline innervation in the limbic system. Brain.

[368] Riddle EL, Fleckenstein AE, Hanson GR (2005). Role of monoamine transporters in mediating psychostimulant effects. AAPS J.

[369] Risbrough VB, Brodkin JD, Geyer MA (2003). GABA-A and 5-HT1A receptor agonists block expression of fear-potentiated startle in mice. Neuropsychopharmacology.

[370] Rosenthal MH, Bryant SL (2004). Benefits of adjunct modafinil in an open-label, pilot study in patients with schizophrenia. Clin. Neuropharmacol.

[371] Roth JD, Rowland NE (1998). Efficacy of administration of dexfenfluramine and pentermine, alone and in combination, on ingestive behavior and body weight in rats. Psychopharmacology (Berl.).

[372] Rüb U, Schultz C, Del Tredici K, Braak H (2001). Early involvement of the tegmentopontine reticular nucleus during the evolution of Alzheimer’s disease-related cytoskeletal pathology. Brain Res.

[373] Rye DB, Jankovic J (2002). Emerging views of dopamine in modulating sleep/wake state from an unlikely source: PD. Neurology.

[374] Sajedianfard J, Khatami S, Semnanian S, Naghdi N, Jorjani M (2005). *in vivo* measurement of noradrenaline in the locus coeruleus of rats during the formalin test a microdialysis study. Eur. J. Pharm.

[375] Sakai K, Salvert D, Touret M, Jouvet M (1977). Afferent connections of the nucleus raphe dorsalis in the cat as visualized by the horseradish peroxidase technique. Brain Res.

[376] Salchner P, Sartori SB, Sinner C, Wigger A, Frank E, Landgraf R, Singewald N (2006). Airjet and FG-7142-induced fos expression differs in rats selectively bred for high and low anxiety-related behavior. Neuropharmacology.

[377] Samuels ER, Hou RH, Langley RW, Szabadi E, Bradshaw CM (2006). Comparison of pramipexole and modafinil on arousal, autonomic, and endocrine functions in healthy volunteers. J. Psychopharmacol.

[378] Samuels ER, Hou RH, Langley RW, Szabadi E, Bradshaw CM (2006). Comparison of amisulpride and pramipexole on alertness, autonomic and endocrine functions in healthy volunteers. Psychopharmacology (Berl.).

[379] Samuels ER, Hou RH, Langley RW, Szabadi E, Bradshaw CM (2007). Modulation of the acoustic startle response by the level of arousal: comparison of clonidine and modafinil in healthy volunteers. Neuropsychopharmacology.

[380] Samuels ER, Hou RH, Langley RW, Szabadi E, Bradshaw CM (2007). Comparison of pramipexole with and without domperidone co- administration on alertness, autonomic, and endocrine functions in healthy volunteers. Br. J. Clin. Pharmacol.

[381] Sands SA, Morilak DA (1999). Expression of α_1D_ adrenergic receptor messenger RNA in oxytocin- and corticotropin-releasing hormone- synthesizing neurons in the rat paraventricular nucleus. Neuroscience.

[382] Sanghera MK, German DC (1983). The effects of benzodiazepine and non-benzodiazepine anxiolytics on locus coeruleus unit activity. J. Neural. Transm.

[383] Saper CB, Scammell TE (2004). Modafinil: A drug in search of a mechanism. Sleep.

[384] Saper CB, Scammell TE, Lu J (2005). Hypothalamic regulation of sleep and circadian rhythms. Nature.

[385] Sasa M, Yoshimura N (1994). Locus coeruleus noradrenergic neurons as a micturition center. Microsc. Res. Tech.

[386] Sasaki M, Shibata E, Tohyama K, Takahashi J, Otsuka K, Tsuchiya K, Takahashi S, Ehara S, Terayama Y, Sakai A (2006). Neuromelanin magnetic resonance imaging of locus coeruleus and substantia nigra in Parkinson’s disease. Neuroreport.

[387] Scammell T, Gerashchenko D, Urade Y, Onoe H, Saper C, Hayaishi O (1998). Activation of ventrolateral preoptic neurons by the somnogen prostaglandin D_2_. Proc. Natl. Acad. Sci. USA.

[388] Scheinin M, Kallio A, Koulu M, Viikari J, Scheinin H (1987). Sedative and cardiovascular effects of medetomidine, a novel selective alpha 2- adrenoceptor agonist, in healthy volunteers. Br. J. Clin. Pharmacol.

[389] Scherder EJ, Luijpen MW, Dijk KR (2003). Activation of the dorsal raphe nucleus and locus coeruleus by transcutaneous electrical nerve stimulation in Alzheimer’s disease: a reconsideration of stimulation- parameters derived from animal studies. Chin. J. Physiol.

[390] Schmid R, Ceurremans P, Luedtke H, Wilhelm BJ, Wilhelm HM (2004). Effect of age on the pupillomotor field. J. Neuroopthalmol.

[391] Scinto LF, Daffner KR, Dressler D, Ransil B, Rentz D, Weintraub S, Mesulam M, Potter H (1994). A potential non-invasive neurobilogical test for Alzheimer’s disease. Science.

[392] Scinto LF, Wu CK, Firla KM, Daffner KR, Saroff D, Geula C (1999). Focal pathology in the Edinger-Westphal nucleus explains pupillary hypersensitivy in Alzheimer’s disease. Acta Neuropathol (Berl).

[393] Senard JM, Arias A, Berlan M, Tran MA, Rascol A, Montastruc JL (1991). Pharmacological evidence of alpha_1_- and alpha_2_-adrenergic supersensitivity in orthostatic hypotension due to spinal cord injury: a case report. Eur. J. Clin. Pharmacol.

[394] Senba E, Matsunaga K, Tohyama M, Noguchi K (1993). Stress-induced c-fos expression in the rat brain: activation mechanism of sympathetic pathway. Brain Res. Bull.

[395] Seutin V, Franchimont N, Massotte L, Dresse A (1990). Comparison of the effect of morphine on locus coeruleus noradrenergic and ventral tegmental area dopaminergic neurons *in vitro*. Life Sci.

[396] Sharpe LG, Pickworth WB (1985). Opposite pupillary size effects in the cat and dog after microinjections of morphine, normorphine and clonidine in the Edinger-Westphal nucleus. Brain Res. Bull.

[397] Shelly MP, Park GR (1984). Morphine toxicity with dilated pupils. Br. Med. J.

[398] Shibasaki T, Tsumori C, Hotta M, Imaki T, Yamanda K, Demura H (1995). The response pattern of noradrenaline release to repeated stress in the hypothalamic paraventricular nucleus differs according to the form of stress in rats. Brain Res.

[399] Shibata E, Sasaki M, Tohyama K, Kanbara Y, Otsuka K, Ehara S, Sakai A (2006). Age-related changes in locus coeruleus on neuromelanin magnetic resonance imaging at 3 tesla. Magn. Reson. Med. Sci.

[400] Shibata E, Sasaki M, Tohyama K, Otsuka K, Sakai A (2007). Reduced signal of locus coeruleus in depression in quantitative neuromelanin magnetic resonance imaging. Neuro Report.

[401] Shih C-D, Chan SHH, Chan JYH (1995). Participation of hypothalamic paraventricular nucleus in locus coeruleus-induced baroreflex suppression in rats. Am. J. Physiol.

[402] Shimizu-Sasamata M, Yamamoto M, Harada M (1993). Cerebral activating properties of indeloxazine HCl and its optical isomers. Pharmacol. Biochem. Behav.

[403] Shirokawa T, Ishida Y, Isobe K (2000). Age-dependent changes in axonal branching of single locus coeruleus neurons projecting to two different terminal fields. J. Neurophysiol.

[404] Shirokawa T, Ishida Y, Isobe K (2000). Changes in electrophysiological properties of axon terminals of locus coeruleus neuron with age in F344 rat. Neurosci. Lett.

[405] Shirokawa T, Ishida Y, Isobe K (2003). Age-related changes in the release and uptake activity of presynaptic axon terminals of rat locus coeruleus neurons. Neurosci. Lett.

[406] Shore PA (1962). Release of serotonin and catecholamines by drugs. Pharmacol. Rev.

[407] Shur E, Checkley S (1982). Pupil studies in depressed patients: an investigation of the mechanism of action of desipramine. Br. J. Psychiatry.

[408] Silva RC, Cruz AP, Avanzi V, Landeira-Fernandez J, Brandao ML (2002). Distinct contributions of median raphe nucleus to contextual fear conditioning and fear-potentiated startle. Neural Plast.

[409] Simson PE, Weiss JM (1987). Alpha-2 receptor blockade increases responsiveness of locus coeruleus neurons to excitatory stimulation. J. Neurosci.

[410] Simson PE, Weiss JM (1989). Peripheral, but not local or intracerebroventricular, administration of benzodiazepines attenuates evoked activity of locus coeruleus neurons. Brain Res.

[411] Singewald N, Kaehler ST, Philippu A (1999). Noradrenaline release in the locus coeruleus of conscious rats is triggered by drugs, stress and blood pressure changes. Neuroreport.

[412] Singewald N, Philippu A (1998). Release of neurotransmitters in the locus coeruleus. Prog. Neurobiol.

[413] Singewald N, Sharp T (2000). Neuroanatomical targets of anxiogenic drugs in the hindbrain as revealed by Fos immunochemistry. Neuroscience.

[414] Sjögren MJ, Hellström PT, Jonsson MA, Runnerstam M, Silander HC, Ben-Menachem E (2002). Cognition-enhancing effect of vagus nerve stimulation in patients with Alzheimer’s disease: a pilot study. J. Clin. Psychiatry.

[415] Smiley JF, Subramanian M, Mesulam MM (1999). Monoaminergic- cholinergic interactions in the primate basal forebrain. Neuroscience.

[416] Smith S.A, Bannister R, Mathias CJ (1992). Pupil function: tests and disorders. Autonomic Failure.

[417] Smith A, Brice C, Nash J, Rich N, Nutt DJ (2003). Caffeine and central noradrenaline: effects on mood, cognitive performance, eye movements and cardiovascular function. J. Psychopharmacol.

[418] Smith MS, Schambra UB, Wilson KH, Page SO, Hulette C, Light AR, Schwinn DA (1995). Alpha 2-adrenergic receptors in human spinal cord: specific localized expression of mRNA encoding alpha 2-adrenergic receptor subtypes at four distinct levels. Brain Res. Mol. Brain Res.

[419] Spencer SE, Sawyer WB, Wada H, Platt KB, Loewy AD (1990). CNS projections to the pterygopalatine parasympathetic preganglionic neurons in the rat: a retrograde transneuronal viral cell body labeling study. Brain Res.

[420] Spencer SJ, Buller KM, Day TA (2005). Medial prefrontal cortex control of the paraventricular hypothalamic nucleus response to psychological stress: possible role of the bed nucleus of the stria terminalis. J. Comp. Neurol.

[421] Spiegel R, DeVos JE (1980). Central effects of guanfacine and clonidine during wakefulness and sleep in healthy subjects. Br. J. Clin. Pharmacol.

[422] Spyer KM, Bannister R, Mathias CJ (1992). Central nervous control of the cardiovascular system. Autonomic Failure.

[423] Sridharan GV, Tallis RC, Leatherbarrow B, Forman WM (1995). A community survey of ptosis of the eyelid and pupil size of elderly people. Age Aging.

[424] Srinivasan J, Schmidt WJ (2003). Potentiation of parkinsonian symptoms by depletion of locus coeruleus noradrenaline in 6-hydroxydopamine- induced partial degeneration of substantia nigra in rats. Eur. J. Neurosci.

[425] Srinivasan J, Schmidt WJ (2004). Behavioral and neurochemical effects of noradrenergic depletions with N-(2-chloroethyl)-N-ethyl-2- bromobenzylamine in 6-hydroxydopamine-induced rat model of Parkinson’s disease. Behav. Brain Res.

[426] Steinhauer SR, Condray R, Kasparek A (2000). Cognitive modulation of midbrain function: task-induced reduction of the pupillary light reflex. Int. J. Psychophysiol.

[427] Steinhauer SR, Siegle GJ, Condray R, Pless M (2004). Sympathetic and parasympathetic innervation of pupillary dilation during sustained processing. Int. J. Psychophysiol.

[428] Sterpenich V, D'Argembeau A, Desseilles M, Balteau E, Albouy G, Vandewalle G, Degueldre C, Luxen A, Collette F, Maquet P (2006). The locus ceruleus is involved in the successful retrieval of emotional memories in humans. J. Neurosci.

[429] Stock G, Rupprecht U, Stumpf H, Schlor KH (1981). Cardiovascular changes during arousal elicited by stimulation of amygdala, hypothalamus and locus coeruleus. J. Auton. Nerv. Syst.

[430] Stone EA, Quartermain D, Lin Y, Lehmann ML (2007). Central α1- adrenergic system in behavioural activity and depression. Biochem. Pharmacol.

[431] Straub RH, Thies U, Kerp L (1992). The pupillary light reflex. 1. Age- dependent and age-independent parameters in normal subjects. Ophthalmologica.

[432] Strange PG (2001). Antipsychotic drugs: importance of dopamine receptors for mechanisms of therapeutic actions and side effects. Pharmacol. Rev.

[433] Strong R, Huang JS, Huang SS, Chung HD, Hale C, Burke WJ (1991). Degeneration of the cholinergic innervation of the locus coeruleus in Alzheimer’s disease. Brain Res.

[434] Sturrock RR, Rao KA (1985). A quantitative histological study of neuronal loss from the locus coeruleus if ageing mice. Neuropathol. Appl. Neurobiol.

[435] Sugiyama H, Hainfellner JA, Schmid-Siegel B, Budka H (1993). Neuroaxonal dystrophy combined with diffuse Lewy body disease in a young adult. Clin. Neuropathol.

[436] Sulser F, Bass AD, Efron DH, Cole JO, Levine J, Wittenborn JR (1968). Pharmacodynamic and biochemical considerations on the mode of action of reserpine-like drugs. Psychopharmacology: A Review of Progress 1957-1967.

[437] Sun MK (1992). Medullospinal vasomotor neurones mediate hypotension from stimulation of prefrontal cortex. J. Auton. Nerv. Syst.

[438] Svensson TH, Almgren O, Dahlof C, Elam M, Engberg G, Hallberg H, Thoren P (1980). Alpha- and beta-adrenoreceptor-mediated control of brain noradrenaline neurons and antihypertensive therapy. Clin. Sci. (Lond.).

[439] Svensson TH, Bunney BS, Aghajanian GK (1975). Inhibition of both noradrenergic and serotonergic neurons in brain by the alpha-adrenergic agonist clonidine. Brain Res.

[440] Swanson LW, Sawchenko PE (1980). Paraventricular nucleus: a site for the integration of neuroendocrine and autonomic mechanisms. Neuroendocrinology.

[441] Swanson LW, Sawchenko PE (1983). Hypothalamic integration: organization of the paraventricular and supraoptic nuclei. Ann. Rev. Neurosci.

[442] Szabadi E (2006). Drugs for sleep disorders: mechanisms and therapeutic prospects. Br. J. Clin. Pharmacol.

[443] Szabadi E, Bradshaw CM, Granville-Grosman K (1988). Biological markers for anxiety state. Recent Advances in Clinical Psychiatry.

[444] Szabadi E, Bradshaw CM (1996). Autonomic pharmacology of α_2_- adrenoceptors. J. Psychopharmacol.

[445] Szabadi E, Bradshaw CM (2000). Mechanisms of action of reboxetine. Rev. Contemp. Pharmacother.

[446] Szabadi E, Tavernor S (1999). Hypo- and hypersalivation induced by psychoactive drugs. Incidence, mechanisms and therapeutic implications. CNS Drugs.

[447] Szabo B, Fröhlich R, Illes P (1996). No evidence for functional imidazoline receptors on locus coeruleus neurons. Naunyn-Schmiedebergs Arch. Pharmacol.

[448] Szot P, White SS, Greenup JL, Leverenz JB, Peskind ER, Raskind MA (2006). Compensatory changes in the noradrenergic nervous system in the locus coeruleus and hippocampus of post-mortem subjects with Alzheimer’s disease and dementia with Lewy bodies. J. Neurosci.

[449] Takimoto GS, Stittsworth JD, Bianchi BR, Stephens JK (1983). Differential sensitivity of hypothalamic norepinephrine and striatal dopamine to catecholamine-depleting agents. J. Pharmacol. Exp. Ther.

[450] Tallarida RJ, Kramer MS, Roy JW, Kester RA, Murray RB, Adler MW (1977). Miosis and fluctuations in the rabbit pupil: effects of morphine and naloxone. J. Pharmacol. Exp. Ther.

[451] Talley EM, Rosin DL, Lee A, Guyenet PG, Lynch KR (1996). Distribution of alpha 2A-adrenergic receptor-like immunoreactivity in the rat central nervous system. J. Comp. Neurol.

[452] Tan EK (2003). Piribedil-induced sleep attacks in Parkinson’s disease. Fundam. Clin. Pharmacol.

[453] Tanaka M, Yoshida M, Emoto H, Ishii H (2000). Noradrenaline systems in the hypothalamus, amygdala and locus coeruleus are involved in the provocation of anxiety: basic studies. Eur. J. Pharmacol.

[454] Tassorelli C, Micieli G, Osipova V, Rossi F, Nappi G (1995). Pupillary and cardiovascular responses to the cold-pressor test. J. Auton. Nerv. Syst.

[455] Tavernor SJ, Abduljawad KA, Langley RW, Bradshaw CM, Szabadi E (2000). Effects of pentagastrin and the cold pressor test on the acoustic startle response and pupillary function in man. J. Psychopharmacol.

[456] Tejani-Butt SM, Ordway GA (1992). Effect of age on [3H]nisoxetine binding to uptake sites for norepinephrine in the locus coeruleus of humans. Brain Res.

[457] Ter Horst GJ, Toes GJ, Van Willigen JD (1991). Locus coeruleus projections to the dorsal motor vagus nucleus in the rat. Neuroscience.

[458] Thase ME, Tran PV, Wiltse C, Pangallo BA, Mallinckrodt C, Detke MJ (2005). Cardiovascular profile of duloxetine, a dual reuptake inhibitor of serotonin and norepinephrine. J. Clin. Psychopharmacol.

[459] Theofilopoulos N, McDade G, Szabadi E, Bradshaw CM (1995). Effects of reboxetine and desipramine on the kinetics of the pupillary light reflex. Br. J. Clin. Pharmacol.

[460] Tomlinson BE, Irving D, Blessed G (1981). Cell loss in the locus coeruleus in senile dementia of Alzheimer’s type. J. Neurol. Sci.

[461] Tsoucaris-Kupfer D, Schmitt H (1972). Hypothermic effect of alpha- sympathomimetic agents and their antagonism by adrenergic and cholinergic blocking drugs. Neuropharmacology.

[462] Tsuruoka M, Matsutani K, Maeda M, Inoue T (2003). Coeruleotrigeminal inhibition of nociceptive processing in the rat trigeminal subnucleus caudalis. Brain Res.

[463] Ulivelli M, Rossi S, Lombardi C, Bartalini S, Rocchi R, Giannini F, Passero S, Battistini N, Lugaresi E (2002). Polysomnographic characterization of pergolide-induced sleep attacks in idiopathic PD. Neurology.

[464] Umemura T, Ueda K, Nishioka K, Hidaka T, Takemoto H, Nakamura S, Jitsuiki D, Soga J, Goto C, Chayama K, Yoshizumi M, Higashi Y (2006). Effects of acute administration of caffeine on vascular function. Am. J. Cardiol.

[465] Unnerstall JR, Kopajtic TA, Kuhar MJ (1984). Distribution of alpha 2 agonist binding sites in the rat and human central nervous system: analysis of some functional, anatomic correlates of the pharmacologic effects of clonidine and related adrenergic agents. Brain Res.

[466] Urade Y, Eguchi N, Qu WM, Sakata M, Huang ZL, Chen JF, Schwarzschild MA, Fink JS, Hayaishi O (2003). Sleep regulation in adenosine A(2A) receptor-deficient mice. Neurology.

[467] Uschakov A, Gong H, McGinty D, Szymusiak R (2006). Sleep-active neurons in the preoptic area project to the hypothalamic paraventricular nucleus and perifornical lateral hypothalamus. Eur. J. Neurosci.

[468] US Modafinil in Narcolepsy Multicentre Study Group (1998). Randomized trial of modafinil for the treatment of pathological somnolence in narcolepsy. Ann. Neurol.

[469] U.S. Modafinil in Narcolepsy Multicentre Study Group (2000). Randomized trial of modafinil as a treatment for the excessive daytime somnolence of narcolepsy. Neurology.

[470] Van Bockstaele EJ, Pieribone VA, Aston-Jones G (1989). Diverse afferents converge on the nucleus paragigantocellularis in the rat ventrolateral medulla: retrograde and anterograde tracing studies. J. Comp. Neurol.

[471] VanderMaelen CP, Aghajanian GK (1980). Intracellular studies showing modulation of facial motoneurone excitability by serotonin. Nature.

[472] van Dongen PA (1981). The central noradrenergic transmission and the locus coeruleus: a review of the data, and their implications for neurotransmission and neuromodulation. Prog. Neurobiol.

[473] van Steveninck AL, van Berckel BN, Schoemaker RC, Breimer DD, van Gerven JM, Cohen AF (1999). The sensitivity of pharmacodynamic tests for the central nervous system effects of drugs on the effects of sleep deprivation. J. Psychopharmacol.

[474] Vayssettes-Courchay C, Bouysset F, Cordi A, Laubie M, Verbeuren TJ (2002). Effects of medullary α_2_-adrenoceptor blockade in the rat. Eur. J. Pharmacol.

[475] Verleye M, Bernet F (1983). Behavioural effects of lesions of the central noradrenergic bundle in the rat. Pharmacol. Biochem. Behav.

[476] Verster JC, Veldhuijzen DS, Volkerts ER (2006). Effects of an opioid (oxycodone/paracetamol) and an NSAID (bromfenac) on driving ability, memory functioning, psychomotor performance, pupil size, and mood. Clin. J. Pain.

[477] Vijayashankar N, Brody H (1979). A quantitative study of the pigmented neurons in the nuclei locus coeruleus and subcoeruleus in man as related to aging. J. Neuropathol. Exp. Neurol.

[478] Voisin DL, Guy N, Chalus M, Dallel R (2005). Nociceptive stimulation activates locus coeruleus neurones projecting to the somatosensory thalamus in the rat. J. Physiol.

[479] Walker DJ, Zacny JP (1998). Subjective, psychomotor, and analgesic effects of oral codeine and morphine in healthy volunteers. Psychopharmacology (Berl.).

[480] Walsh JK, Randazzo AC, Stone KL, Schweitzer PK (2004). Modafinil improves alertness, vigilance, and executive function during simulated night shifts. Sleep.

[481] Walter H, Lesch OM, Stohr H, Grunberger J, Gutierrez-Lobos K (2005). Reaction to pain stimulus before and during hypnosis measured by pupillary reaction. Am. J. Clin. Hypn.

[482] Ward DG, Gunn CG (1976). Locus coeruleus complex: elicitation of a pressor response and a brain stem region necessary for its occurrence. Brain Res.

[483] Warren JB, Dollery CT, Fuller RW, Williams VC, Gertz BJ (1989). Assessment of MK-912, an α_2_-adrenoceptor antagonist, with use of intravenous clonidine. Clin. Pharmacol. Ther.

[484] Watson CJ, Lydic R, Baghdoyan HA (2007). Sleep and GABA levels in the oral part of rat pontine reticular formation are decreased by local and systemic administration of morphine. Neuroscience.

[485] Webster L, Andrews M, Stoddard G (2003). Modafinil treatment of opioid-induced sedation. Pain Med.

[486] Weerasuriya K, Shaw E, Turner P (1984). Preliminary clinical pharmacological studies of S3341, a new hypotensive agent, and comparison with clonidine in normal males. Eur. J. Clin. Pharmacol.

[487] Werling LL, Brown SR, Cox BM (1987). Opioid receptor regulation of the release of norepinephrine in brain. Neuropharmacology.

[488] Wesensten NJ, Killgore WDS, Balkin TJ (2005). Performance and alertness of caffeine, dextroamphetamine, and modafinil during sleep deprivation. J. Sleep Res.

[489] Westlund KN, Bowker RM, Ziegler MG, Coulter JD (1983). Noradrenergic projections to the spinal cord of the rat. Brain Res.

[490] Westlund KN, Coulter JD (1980). Descending projections of the locus coeruleus and subcoeruleus/medial parabrachial nuclei in monkey: axonal transport studies and dopamine-beta-hydroxylase immunocytochemistry. Brain Res.

[491] White SR, Fung SJ, Barnes CD (1991). Norepinephrine effects on spinal motoneurons. Prog. Brain Res.

[492] White SM, Lambe CJT (2003). The pathophysiology of cocaine abuse. J. Clin. Forensic Med.

[493] Wilhelm B, Giedke H, L?dtke H, Bittner E, Hofmann A, Wilhelm H (2001). Daytime variations in central nervous system activation measured by a pupillographic sleepiness test. J. Sleep Res.

[494] Williams JT, Henderson G, North RA (1985). Characterization of alpha 2-adrenoceptors which increase potassium conductance in rat locus coeruleus neurones. Neuroscience.

[495] Williams J, Lacey M (1988). Actions of cocaine on central monoamine neurons: intracellular recordings *in vitro*. NIDA Res. Monogr.

[496] Williams JT, North RA (1985). catecholamine inhibition of calcium action potentials in rat locus coeruleus neurones. Neuroscience.

[497] Wilson MC, Bedford JA, Buelke J, Kibbe AH (1976). Acute pharmacological activity of intravenous cocaine in the rhesus monkey. Psychopharmacol. Commun.

[498] Winslow JT, Parr LA, Davis M (2002). Acoustic startle, prepulse inhibition, and fear-potentiated startle measured in rhesus monkeys. Biol. Psychiatry.

[499] Wisor JP, Nishino S, Sora I, Uhl GH, Mignot E, Edgar DM (2001). Dopaminergic role in stimulant-induced wakefulness. J. Neurosci.

[500] Wright KP Jr, Badia P, Myers BL, Plenzler SC, Hakel M (1997). Caffeine and light effects on nighttime melatonin and temperature levels in sleep-deprived humans. Brain Res.

[501] Yaïci E-D, Rampin O, Calas A, Jestin A, McKenna KE, Leclerc P, Benoit G, Giuliano F (2002). α2a and α2c adrenoceptors on spinal neurons controlling penile erection. Neuroscience.

[502] Yaksh TL, Provencher JC, Rathbun ML, Myers RR, Powell H, Richter P, Kohn FR (2000). Safety assessment of encapsulated morphine delivered epidurally in a sustained-release multivesicular liposome preparation in dogs. Drug Deliv.

[503] Yang Y, Beyreuther K, Schmitt HP (1999). Spatial analysis of the neuronal density of aminergic brainstem nuclei in primary neurodegenerative and vascular dementia: a comparative immunocytochemical and quantitative study using a graph method. Anal. Cell Pathol.

[504] Yang LL, Niemann CU, Larson MD (2003). Mechanism of pupillary reflex dilation in awake volunteers and in organ donors. Anesthesiology.

[505] Yeomans JS, Frankland PW (1996). The acoustic startle reflex: neurons and connections. Brain Res. Brain Res. Rev.

[506] Yoshimura N, Sasa M, Yoshida O, Takaori S (1990). Alpha 1- adrenergic receptor-mediated excitation from the locus coeruleus of the sacral parasympathetic preganglionic neuron. Life Sci.

[507] Yoss RE, Moyer NJ, Hollenhurst RW (1970). Pupil size and spontaneous pupillary waves associated with alertness, drowsiness, and sleep. Neurology.

[508] Yuan L, Brewer C, Pfaff D (2002). Immediate-early Fos protein levels in brainstem neurons of male and female gonadectomized mice subjected to cold exposure. Stress.

[509] Zaborszky L, Cullinan WE, Luine VN (1993). Catecholamine-cholinergic interaction in the basal forebrain. Prog. Brain Res.

[510] Zaccara G, Gangemi PF, Muscas GC, Paganini M, Pallanti A, Parigi A, Messori A, Arnetoli G (1992). Smooth-pursuit eye movements: alterations in Alzheimer’s disease. J. Neurol. Sci.

[511] Zacny E (1982). The role of alpha-2-adrenoceptors in the hypothermic effect of clonidine in the rat. J. Pharm. Pharmacol.

[512] Zacny JP (2005). Profiling the subjective, psychomotor, and physiological effects of tramadol in recreational drug users. Drug Alcohol Depend.

[513] Zamir N, Palkovits M, Brownstein MJ (1984). The distribution of immunoreactive α-neo-endorphin in the central nervous system of the rat. J. Neurosci.

[514] Zarow C, Lyness SA, Mortimer JA, Chui HC (2003). Neuronal loss is greater in the locus coeruleus than nucleus basalis and substantia nigra in Alzheimer and Parkinson diseases. Arch. Neurol.

[515] Zecca L, Tampellini D, Gerlach M, Riederer P, Fariello RG, Sulzer D (2001). Substantia nigra neuromelanin: structure, synthesis, and molecular behaviour. J. Clin. Pathol. Mol. Pathol.

[516] Zheng G, Dwoskin LP, Crooks PA (2006). Vesicular monoamine transporter 2: role as a novel target for drug development. AAPS J.

[517] Zhou J (2004). Norepinephrine transporter inhibitors and their therapeutic potential. Drugs Future.

[518] Zhu H, Zhou W (2001). Morphine induces synchronous oscillatory discharges in the rat locus coeruleus. J. Neurosci.

[519] Zucca FA, Bellei C, Giannelli S, Terreni MR, Gallorini M, Rizzio E, Pezzoli G, Albertini A, Zecca L (2006). Neuromelanin and iron in human locus coeruleus and substantia nigra during aging: consequences for neuronal vulnerability. J. Neural Transm.

[520] Zweig RM, Cardillo JE, Cohen M, Giere S, Hedreen JC (1993). The locus coeruleus and dementia in Parkinson’s disease. Neurology.

[521] Zweig RM, Ross CA, Hedreen JC, Peyser C, Cardillo JE, Folstein SE, Price DL (1992). Locus coeruleus involvement in Huntington’s disease. Arch. Neurol.

[522] Zwyghuizen-Doorenbos A, Roehrs TA, Lipschutz L, Timms V, Roth T (1990). Effects of caffeine on alertness. Psychopharmacology (Berl.).

